# Investigations of spin textures at small length and short timescales with neutron, x-ray, and optical methods

**DOI:** 10.1126/sciadv.aef3254

**Published:** 2026-09-16

**Authors:** Peter Fischer, Collin Broholm, Charles C. Tam, Stephen D. Wilson, Theo Rasing, Alexey V. Kimel

**Affiliations:** ^1^ Materials Sciences Division, Lawrence Berkeley National Laboratory, Berkeley, CA 94720, USA.; ^2^ Physics Department, University of California Santa Cruz, Santa Cruz, CA 95064, USA.; ^3^ William H. Miller III Department of Physics and Astronomy and, Institute for Quantum Matter, Johns Hopkins University, Baltimore, MD 21218, USA.; ^4^ Materials Department, University of California Santa Barbara, Santa Barbara, CA 93106, USA.; ^5^ Radboud University Nijmegen, Institute for Molecules and Materials, Heyendaalseweg 135, 6525 AJ Nijmegen, Netherlands.

## Abstract

The theoretical prediction, experimental discovery, and validation of the electron spin 100 years ago have provided the scientific foundation toward a fundamental understanding of magnetism. The interactions among spins and their arrangements into spin textures at the microscopic scale and their dynamical behavior across multiple timescales largely determine the properties and behavior of magnetic materials and their functionalities in technological applications. Experimental investigations of spin textures at scientifically and technologically relevant small length and ultrashort timescales, down to nanometers and femtoseconds, respectively, have stimulated and benefited from advances in developing experimental probes using neutrons, x-rays, and optical methods. On the occasion of the centennial anniversary of the electron spin, here, we review pioneering and groundbreaking scientific accomplishments with these methods, describe the current state of the art, and provide a perspective into future directions, such as quantum information systems or neuromorphic computing, which leverage the coherence and correlations between electron spins.

## INTRODUCTION

Although magnetic materials have been known to humans and have been used in technological applications for thousands of years, the prediction and discovery of the spin of the electrons lastly provided the fundamental framework for developing a scientific understanding of magnetic materials. The interaction between magnetic moments, their arrangements into microscopic domains, and their dynamical response ultimately determines the properties, behavior, and functionality of magnetic materials and devices built from those.

Although there are only a few naturally occurring ferromagnets, the concept of Heisenberg exchange interaction among two spins also led to the prediction and discovery of antiferromagnetic materials, which are even more abundant in nature than FMs. The fact that they only weakly respond to applied external fields owing to their net zero magnetic moment has mostly kept them hidden until Néel’s proposal in the 1930s. Initially considered by Néel to be “interesting but useless,” antiferromagnetic materials play an ever-increasing role, e.g., in the phenomenon of exchange bias, and, since the discovery of giant magnetoresistance in 1988, which was awarded to the Nobel Prize to A. Fert and P. Grünberg in 2007, also in many technological applications. The recent prediction of altermagnetic materials, which originated from fundamental symmetry considerations, is another example of how the spin of the electron and its atomic scale properties continue to drive future scientific and technological developments.

These scientific discoveries have opened up a completely new approach to computation, data storage, and sensor technologies. So far, semiconductor technologies that have started with the concept of a transistor in the 1950s to the most advanced complementary metal-oxide semiconductor technologies as of today dominate a global multitrillion industry, which is critical to enable the information technologies we are relying on today and even more in the future. The grand challenge is that the fundamental concept of using the charge of the electron as the fundamental unit in those devices requires an enormous amount of energy with inevitable and substantial losses in power due to inevitable ohm resistance in nonsuperconducting materials. An obvious alternative is to build technologies using the spin of the electron only, which would allow orders of magnitude less energy and which is coined as spintronics.

Advances in theoretical and experimental efforts are essential for making progress in this research and technology areas. Probing those spin textures at small lengths and fast timescales with adequate spatial and temporal resolution is essential and requires the most exquisite instrumental tools. Probing magnetic materials with neutrons, x-rays, and optical methods has proven to be a very valuable approach. On the occasion of the centennial of the discovery of the electron spin, we provide a short review of groundbreaking achievements, current state of the art capabilities, and a perspective into the future of those capabilities to deepen our understanding of the role of the spin in materials, the discovery of novel phenomena often relying on artificially designed unconventional magnetic materials, and its exploitation in future critical technologies.

## NEUTRON SCATTERING STUDIES OF QUANTUM MAGNETISM

### Neutron scattering: A versatile probe of static and dynamic magnetism

A neutral fermion (i) with a finite magnetic dipole moment well below that of the electron, (ii) with a de Broglie wavelength similar to the electronic spin-spin spacing, and (iii) with a kinetic energy similar to the interaction energy in magnetic solids would furnish an ideal probe of magnetism. It is fortunate that these are characteristics of free neutrons [$\mu_{n}=\gamma \left(m_{e}/m_{n}\right)\mu_{B}=-1.042\times 10^{-3}\mu_{B}$ and $\lambda_{n}=h/\sqrt{2m_{n}E}=9.045$ Å$\cdot {\text{meV}}^{1/2}/\sqrt{E}$] and that neutrons have an appreciable lifetime (878 s) and can be extracted from nuclei to form neutron beams.

The potential of neutron beams to probe magnetism through their electromagnetic interaction with the spin and orbital dipole moments of electrons was recognized and demonstrated by C. G. Shull and B. N. Brockhouse in the early 1950s. For developing and pioneering neutron scattering techniques, Brockhouse and Shull were awarded the 1994 Nobel Prize in Physics (*1*).

A defining characteristic of neutrons as a probe of condensed matter and magnetism in particular is the fact that the interactions are sufficiently weak that an accurate description of their scattering from condensed matter is obtained within the Born approximation. This enables a close quantitative engagement between theory and neutron scattering experiments through the partial differential scattering cross section, which for magnetic scattering takes the form (*2*)

$$\frac{d^{2}\sigma }{d\Omega {\mathrm{dE}}_{f}}=\frac{k_{f}}{k_{i}}{\left(\gamma r_{0}\right)}^{2}{\left|\frac{g}{2}F\left(Q\right)\right|}^{2}\sum_{\alpha \beta }\left(\delta_{\alpha \beta }-{\hat{Q}}_{\alpha }{\hat{Q}}_{\beta }\right)S^{\alpha \beta }\left(Q,\omega \right)$$ (1)

Here, $Q=k_{i}-k_{f}$ and $\hbar \omega =E_{i}-E_{f}$ is the wave vector and energy transfer to the sample, *g* is the Lande factor, *F*(*Q*) is the magnetic form factor, the Fourier transform of the atomic spin density distribution, $r_{0}=2.818$ fm is the classical electron radius, and γ=−1.913 is the gyromagnetic ratio for the neutron. The overall strength of magnetic scattering is thus set by the magnetic scattering length $\gamma r_{0}=-5.391$ fm. Because this number is similar in magnitude to nuclear scattering lengths, magnetic neutron scattering is readily detected in the presence of the accompanying nuclear scattering (*3*). This is another fortuitous feature that helps to explain why Brockhouse, in talks, lightheartedly would say, “If the neutron did not exist, it would need to be invented.”

The key part of the scattering cross section is the dynamic two-point correlation function between electron spin at different locations **r**, **r**′, and at times separated by *t*

$$S^{\alpha \beta }\left(Q,\omega \right)=\frac{1}{2\pi \hbar }\int_{-\infty }^{\infty }{\mathrm{dte}}^{-\mathrm{i\omega t}}\sum_{rr^{\prime }}e^{iQ\cdot \left(r-r^{\prime }\right)}\langle S_{r}^{\alpha }\left(t\right)S_{r^{\prime }}^{\beta }\left(0\right)\rangle$$ (2)

The summation in Eq. 1 expresses the anisotropic form of the dipole interaction between the neutron and electron dipole moments, which makes neutron scattering sensitive only to the component of electron spin that lies in the normal plane to wave vector transfer **Q**.

Time-independent spin correlations give rise to elastic magnetic scattering, a term proportional to δ(ω) in Eq. 1. For commensurate periodic structures, the corresponding elastic cross section takes the form (*2*)

$${\left(\frac{\mathrm{d\sigma}}{d\Omega }\right)}_{\text{el}}=N_{m}\frac{{\left(2\pi \right)}^{3}}{v_{m}}\sum_{G_{m}}{\left|F_{M}^{⊥}\left(Q\right)\right|}^{2}\delta \left(Q-G_{m}\right)$$ (3)

where $N_{m}$ is the number of magnetic unit cells in the sample, $v_{m}$ is the volume of the magnetic unit cell, and $G_{m}$ are the set of all magnetic reciprocal lattice vectors. The magnetic vector structure factor takes the form

$$F_{M}\left(Q\right)=\frac{\gamma r_{0}}{2}\sum_{j=1}^{n}f_{j}\left(Q\right)\mu_{j}e^{iQ\cdot r_{j}}e^{-\frac{1}{2}\langle u_{j}^{2}\rangle Q^{2}}$$ (4)

Here, the summation is over the *n* magnetic atoms at positions $r_{j}$ in the magnetic unit cell with form factor $f_{j}\left(Q\right)$ and mean square displacements $\langle u_{j}^{2}\rangle$. The ordered moment vector in units of the Bohr magneton is **μ***_j_*. Regarding inelastic scattering, only spin components perpendicular to $\hat{Q}$ contribute to magnetic diffraction in Eq. 3: $F_{M}^{⊥}\left(Q\right)=\hat{Q}\times \left(F_{M}\left(Q\right)\times \hat{Q}\right)$.

The quantitative information about static and dynamic spin correlations that neutron scattering provides has been key to advancing our understanding of magnetism on energy scales from nano–electron volts to electron volts and length scales from picometers to micrometers. In the following, we shall illustrate the role of magnetic neutron scattering in quantum materials science and indicate promising new directions through a couple of examples.

### Local and long-range spin order

Neutron scattering continues to reveal an astonishing variety of spin textures in solids. The complexity of magnetic interactions and their interplay with crystal symmetry make this a rich domain in fundamental condensed matter physics with myriad opportunities for applications. A recent focus on **k** = 0 antiferromagnets where uniform magnetization is not forbidden by symmetry (*4*) has increased interest in accurate information about spin structures through neutron diffraction.

It is fortunate that highly efficient instrumentation at spallation neutron sources can now provide this information even for a ∼1-mg single crystal or a ∼100-mg powder sample. Symmetry analysis is key to determining magnetic order from diffraction data, as phase information is lost in the diffraction cross section (Eq. 3) so that direct Fourier inversion is not possible. In addition to tabular information about magnetic space groups, the Bilbao Crystallographic server (https://cryst.ehu.es/cryst/) contains descriptions of more than 2000 experimentally determined long-range ordered magnetic structures (https://cryst.ehu.es/magndata/) (*5*).

Competing interactions can give rise to modulated magnetic structures, which are “incommensurate” in the sense that the magnetic wave vector **k** cannot be expressed as a rational fraction of a reciprocal lattice vector. This implies magnetic satellite Bragg peaks that surround reciprocal lattice points associated with the chemical structure $G_{m}=G\pm k$. Curiously, this facilitates the determination of the ordered structure because magnetic diffraction is separated from nuclear diffraction, which decouples nuclear and magnetic refinement and reduces problems from multiple scattering and extinction.

**Fig. 1.**
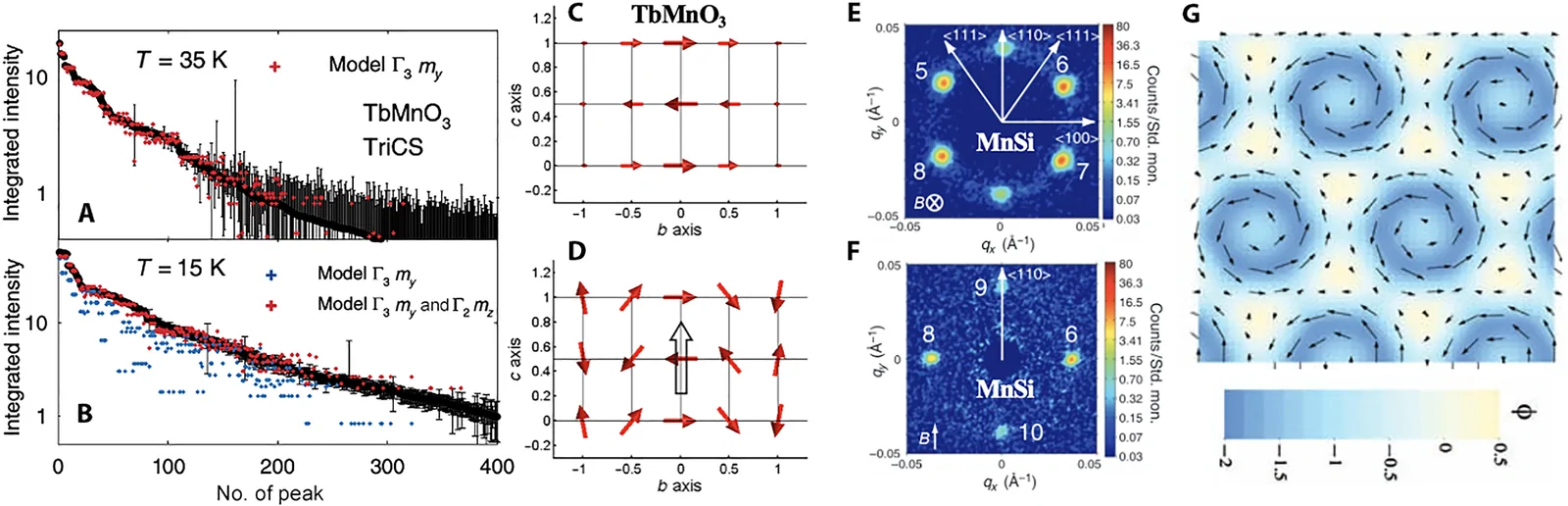
Determination of long-range ordered spin structures at different length scales with neutron diffraction. (**A** and **B**) Magnetic Bragg intensities from two different phases of orthorhombic TbMnO_3_ with the corresponding inferred incommensurate spin structures in (**C**) and (**D**). Reprinted with permission from (*7*). Copyright (2005) by the American Physical Society. (**E** and **F**) Small-angle neutron diffraction from MnSi in the skyrmion phase at *T* = 27.7 K with field applied along the <110> direction of the cubic inversion symmetry breaking structure. A field of 0.16 T is perpendicular to the scattering plane in (E) while a field of 0.19 T is vertical in (F) with the low symmetry horizontal axis containing peaks 6 and 8 from frame (E). (**G**) Inferred skyrmion lattice structure that has a finite integrated skyrmion density per unit cell Φ=−1. Frames (E to G) were reproduced/modified.

An example of incommensurate magnetic order is provided by the insulating orthorhombic distorted perovskite antiferromagnet TbMnO_3_. This material was identified as multiferroic in that magnetic order and ferroelectric polarization coexist and interact (*6*). Peaks in the specific heat capacity indicate three magnetic phase transitions at $T_{c1}=42$ K, $T_{c2}=27$ K, and $T_{c3}=7$ K. Figure 1A shows the integrated intensity of more than 900 magnetic Bragg peaks acquired in the highest-temperature ordered state at *T* = 35 K where $k=0.27b^{*}$ (*7*). The longitudinally modulated incommensurate spin configuration shown in Fig. 1C is associated with the irreducible representation $\Gamma_{3}$, and the calculated diffraction (red points) provides a satisfactory account of the neutron diffraction data (black points). For $T=15K\in \left[T_{c2},T_{c3}\right]$, the blue points calculated for the longitudinal incommensurate structure do not describe the measured black points. Adding in a transverse modulation polarized along the c axis (Fig. 1D) with the symmetry of irreducible representation $\Gamma_{3}$, however, yields an excellent fit (red compared to black points in Fig. 1B). This structure breaks inversion symmetry, as can readily be appreciated by inspecting Fig. 1D. It follows that electrically insulating TbMnO_3_ must have ferroelectric polarization along c in the transverse modulated incommensurate phase, as is found in pyrocurrent measurements (*6*).

Competing interactions also drive incommensurate magnetism in metallic MnSi (*8*). The material is nearly ferromagnetic, but the structure breaks inversion symmetry so that Dzyaloshinskii-Moriya interactions of the form $D\cdot S_{i}\times S_{j}$ are allowed and compete with ferromagnetic interactions $-|J|S_{i}\cdot S_{j}$ to produce a long wavelength–modulated structure. The cubic nature of the B20 structure further ensures that the energy scale favoring the <111> orientation of the magnetic wave vector is much smaller than *J* and *D*. Small-angle neutron scattering (SANS) was key to determining the long-wavelength structures of MnSi (*8*). This was particularly the case for the so called A phase that exists in a small pocket in the field temperature phase diagram for small applied fields (≈0.16 T) at temperatures T≈27.7 K just below the transition to magnetic order. Figure 1E shows SANS data for fields applied along the incident beam, and it shows a hexagonal pattern of magnetic Bragg peaks in the plane Q⊥B. A similar pattern of six magnetic Bragg peaks was consistently found in the normal plane to **B** independent of the crystal orientation (see Fig. 1F where that plane is perpendicular to the page), and this is evidence of the two-dimensional (2D) Skyrmion lattice in Fig. 1G.

**Fig. 2.**
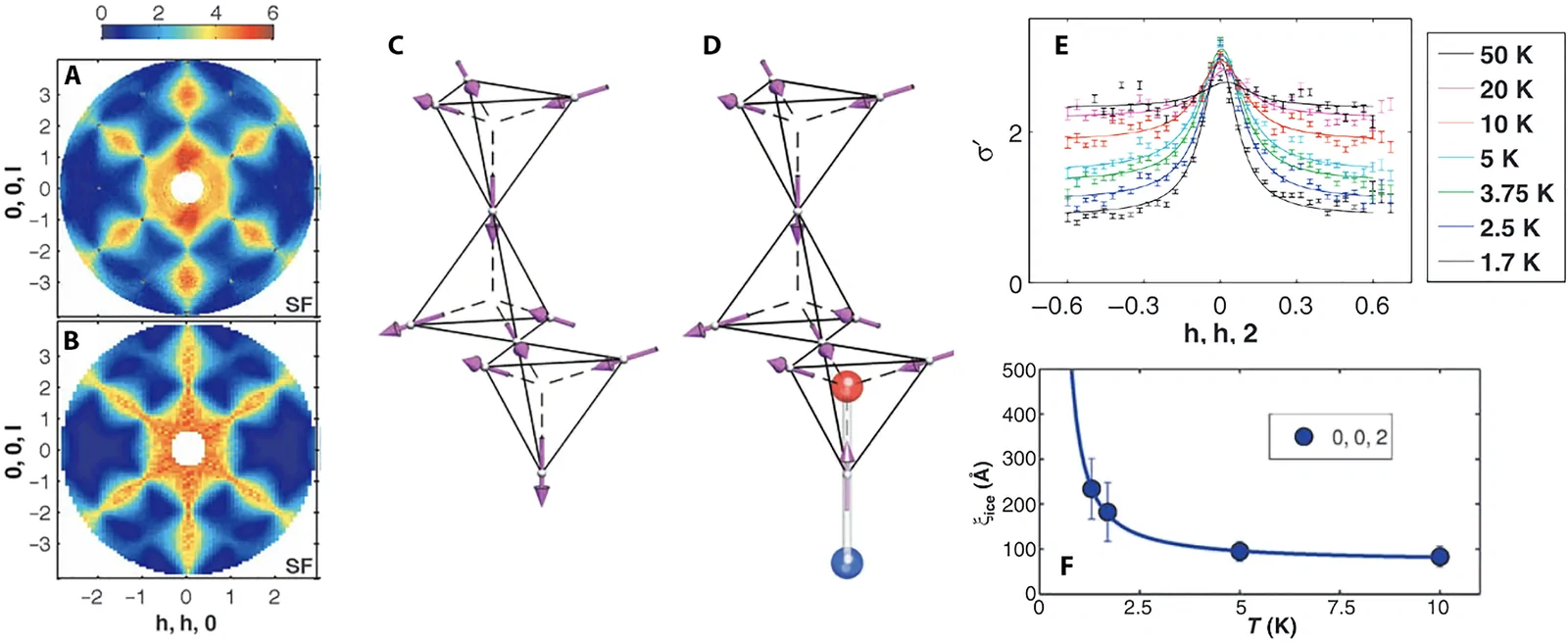
Neutron spin flip diffraction from Ho_2_Ti_2_O_7_ measured at *T* = 1.7 K. The diffuse pattern detected in (**A**) shows no Bragg peaks but sharp pinch points. The calculated diffuse scattering in (**B**) is for spin ice order where every tetrahedron has two spins pointing in an two spins pointing out as in (**C**). Flipping any spin from this state creates a pair of oppositely charged magnetic monopolar defect as shown in (**D**). (**E**) Spin-Flip scattering at increasing temperatures. The width of the pinch point measured in an (*h, h*, 2) scan is sensitive to the *T-*dependent spacing between monopoles reported in (**F**). Figure was reproduced/modified from the American Physical Society with permission.

Although long-range magnetic order is the norm for $k_{B}T\ll J$, there are interesting exceptions. In the pyrochlore lattice of corner-sharing tetrahedra (*9*), rare-earth spin-orbital moments are constrained by the crystal electric field (CEF) to point either into or out from the center of the tetrahedra (Fig. 2C). Short-range exchange and dipolar interactions favor divergence-free spin configurations, where as many moments point in as point out from every tetrahedron. The “spin ice” manifold of states that satisfies this constraint is macroscopic carrying the Pauling entropy $S_{0}=\frac{1}{2}R\text{log}\left(\frac{3}{2}\right)$, which means that there are no long-range spin order and no magnetic Bragg peaks. The spin-flip component of the magnetic neutron diffraction was measured at low T=1.7K by Fennell *et al.* (*10*), and there are no magnetic Bragg peaks (Fig. 2A). The coherent diffuse scattering that takes their place is perfectly accounted for by the two-in-two-out spin ice manifold, including the pinch point regions whose sharpness reflect the long distance between tetrahedra that violate the two-in-two-out rule.

Figure 2D shows that a spin flip within the spin ice manifold generates two defect tetrahedra, where more spins point in (red) or out from (blue) the center. The inverse width of the pinch point in an (*h, h,* 2) scan (Fig. 2E) is the mean spacing between defect tetrahedra and can be seen to grow upon cooling. The defect tetrahedra may be associated with magnetic monopoles of opposite sign, and spin ice physics can alternatively be described in terms of monopoles on the diamond lattice with Coulomb interactions (*11*). We shall discuss time-resolved neutron diffraction experiments that probe the very slow dynamics of such monopoles in the “Magnetic scattering from driven spin systems” section.

### Magnons and spinons in quantum magnets

Inelastic neutron scattering provides exquisite access to magnetic excitations in interacting spin systems. Simple 3D nonfrustrated spin system generally have spin-wave excitations that give rise to poles in the dynamic spin correlation function (Eq. 2) when ℏω=ϵ(Q). These correspond to neutrons being scattered by creating (ℏω>0) or annihilating (ℏω<0) a magnon with a dispersion relation ϵ(Q). Measurements of these dispersion relations in insulators (*12*) and metals (*13*) can be used to experimentally determine magnetic interactions.

**Fig. 3.**
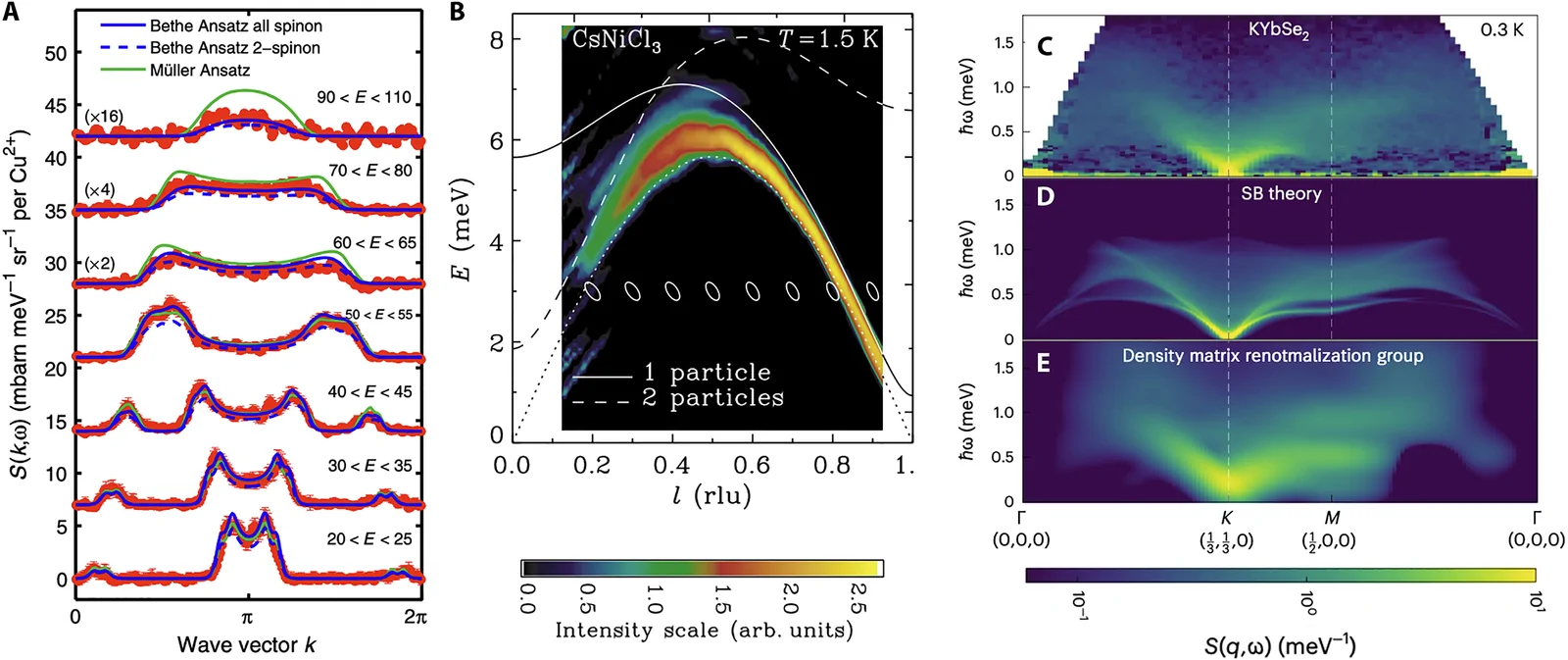
Neutron magnon and spinon scattering. (**A**) Neutron-multispinon scattering in the spin-1/2 chain compound KCuF_3_. Data shown are constant energy transfer cuts through the magnetic neutron spin flip scattering cross section along the spin-chain wave vector transfer axis. Comparison is made to theory as described in the text. Reprinted with permission from (*294*). Copyright (2013) by the American Physical Society. (**B**) Neutron scattering from magnons in the Haldane spin-1 chain compound CsNiCl_3_. A threefold degenerate magnon acquires damping when magnon decay into two magnons is kinematically allowed. Reprinted with permission from (*21*). Copyright (2001) by the American Physical Society. (**C**) Magnetic neutron scattering from the spin-1/2 triangular lattice antiferromagnet KYbSe_2_ showing predominantly continuum scattering despite the presence of long-range magnetic order. (**D** and **E**) Theoretical calculations of the dynamic spin correlation function as described in the text. Reproduced from (*24*) with permission from SNCSC.

In low-dimensional and frustrated quantum magnets, the magnetic excitations can take radically different forms, and this is reflected in qualitatively different spin correlation functions S(Qω). Introduced as a simple model of magnetism by Bethe (*14*) shortly after the advent of quantum mechanics, it is remarkable that there are now many faithful experimental realizations of the antiferromagnetic Heisenberg spin-1/2 chain (*15*). Figure 3A shows constant energy cuts through S(Qω) for one of these systems, KCuF_3_. Rather than a series of sharp peaks that would correspond to cutting through magnon dispersion relations, there is clear evidence of continuum scattering in the broad regions of wave vector transfer where S(Qω) is finite. The data are compared to an early phenomenological theory by Müller *et al.* (*16*) and to the exact two- spinon (*17*) and multispinon (*18*) contributions to the dynamic spin correlation function. The remarkable agreement between experiment and data for this exactly solvable model indicates the excellent quantitative understanding both of the magnetism of KCuF_3_ and of the magnetic neutron scattering process. It also confirms that magnetic materials exist in which the fundamental excitations are not spin-1 magnons but spin-1/2 spinons that form a Luttinger liquid with gapless excitations and a *T* linear–specific heat capacity.

As theoretically shown by Haldane (*19*), the spin-1 antiferromagnetic spin chain is completely different. The ground state is a valence bond solid (*20*), and there is a gap in the magnetic excitation spectrum and above that a triplet of spin-1 magnons. Thirty-five years ago at the Aspen Center for Physics, one of the authors (Broholm) attended an informal presentation by Haldane where he sketched the characteristic excitation spectrum that he anticipated for the spin-1 chain. Figure 3B shows the results of a neutron scattering experiment on CsNiCl_3_ by Zaliznyak *et al.* (*21*), which displays the salient characteristics predicted by Haldane. There is a coherent magnon excitation above a spin gap with a dispersion relation that is not symmetric about q=π/2 (*l* = 0.5), which reflects the lack of long-range antiferromagnetic order. This implies that the magnon can merge with the lower edge of its two-particle continuum (dashed line in Fig. 3B). Other theoretically predicted features of the Haldane spin chain such as topologically protected spin-1/2 degrees of freedom at chain ends (*20*) were also verified through magnetic neutron scattering (*22*).

In 1973, Anderson (*23*) introduced the concept of a resonating valence bond state and showed that it is energetically competitive as a variational ground state for the spin-1/2 Heisenberg antiferromagnet on the triangular lattice. While the simple triangular lattice model was subsequently found to have 120 ° three sublattice antiferromagnetic order, it was later shown that extensions of the model including second–nearest neighbor AFM interactions can destabilize the 120° structure and favor the RVB state. Progress in neutron spectroscopy and the discovery of highly 2D triangular lattice AFM have demonstrated that even in spin systems with static long-range magnetic order, magnetic excitations can take the form expected for the RVB state. Figure 3C shows that spin excitations in the rare-earth delafossite triangular lattice spin-1/2 AFM form a gapless continuum (*24*), resembling a 2D version of the multispinon continuum of the spin-1/2 chain (Fig. 3A). Although there is no exact theory for the 2D triangular lattice case, both a Schwinger boson mean field theory and the numerical density matrix renormalization group calculation reproduce salient features observed experimentally (*24*).

### Magnetic scattering from driven spin systems

So far, we have discussed the use of magnetic neutron scattering to probe magnetic structures and excitations in thermal equilibrium. However, more often than not, applications of magnetic materials involve subjecting these to time-dependent external fields or current densities that drive them beyond thermal equilibrium. High-efficiency neutron instrumentation offers the possibility to probe magnetism in driven systems. These “in operando” experiments are not only needed to optimize materials for applications but can also provide critical insights to advance fundamental understanding of magnetism.

The experiments exploit event mode neutron detection and/or pulsed neutron beams coordinated with time-dependent perturbations of the sample. For “rare” perturbations such as intense magnetic field pulses, where the sample relaxes long before the next pulse can be provided, the experiments are best conducted at pulsed spallation neutron sources to maximize neutron flux during the perturbation. These experiments are conducted by controlling the time delay between the periodic neutron pulse and the pulsed sample environment. The ultimate time resolution is given by the pulse width of the neutron Δt≈10 μs. For perturbations that can be applied as soon as the material has recovered from the prior pulse, the figure of merit is the time average neutron flux, which favors reactor-based instrumentation. Here, neutrons arrive at random, and the neutron detection time is used to determine when each neutron interacted with the sample. In this case, the ultimate time resolution Δt≈10 μs is set by the distribution in the neutron transit time from sample to detector. Although the time resolution is fairly coarse, virtually, any neutron scattering experiment with event mode detection can be retrofitted to add a sample time dimension to the data.

**Fig. 4.**
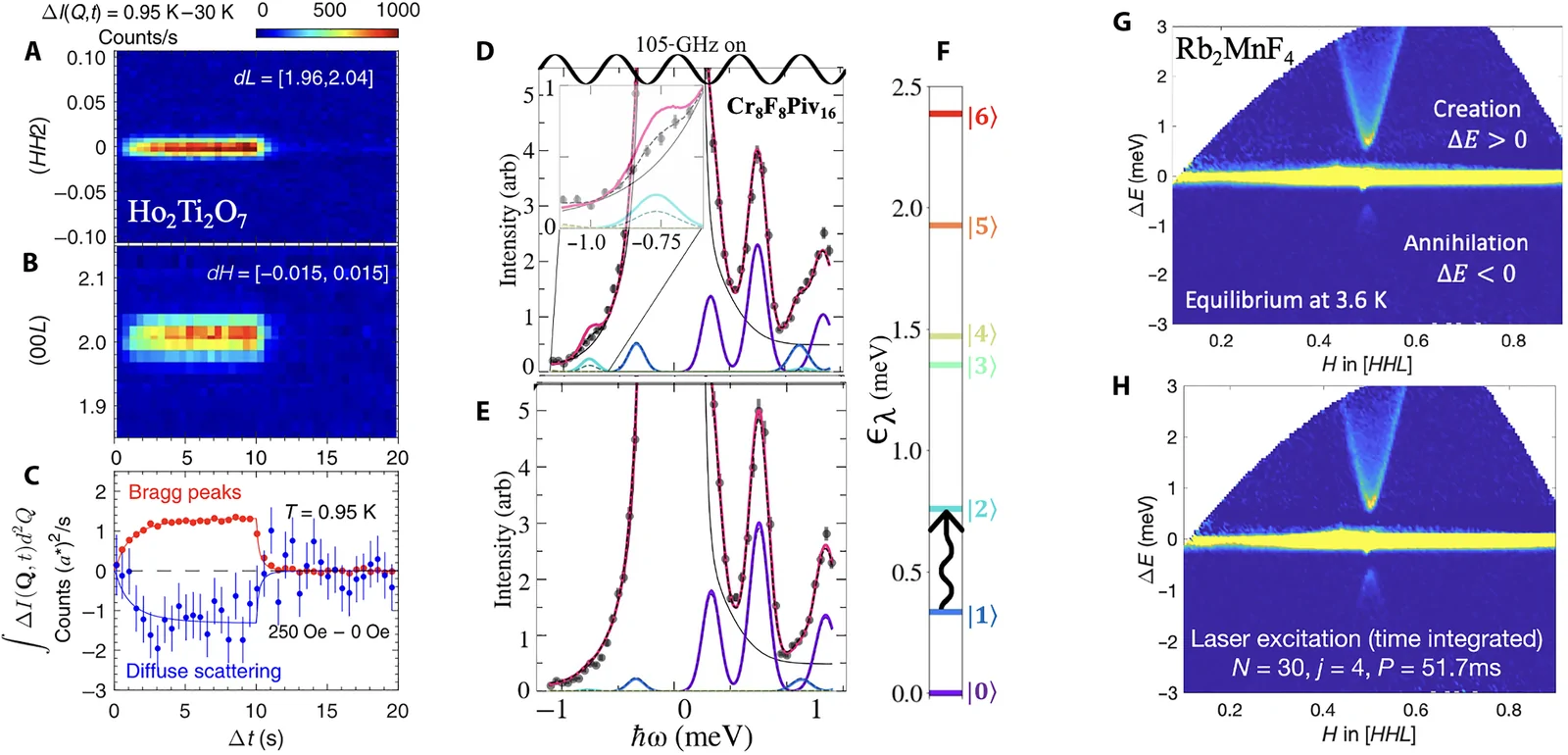
Magnetic neutron scattering from spin systems driven by stepped DC magnetic fields, resonant microwaves, and laser pulses. (**A** and **C**) Time-resolved magnetic neutron diffraction from the spin ice material Ho_2_Ti_2_O_7_ at *T* = 0.95 K while toggling a 250-Oe field on and off in 10-s steps. The appearance of the (002) Bragg peak indicates magnetic monopoles of opposite charge are driven apart. (C) Diffuse magnetic scattering is correspondingly reduced when the field is on. Frames (A and C) were reproduced/modified from (*25*). (**D** and **E**) Inelastic magnetic neutron scattering from Cr_8_F_8_Piv_16_ at T=2.3(1) K and $\mu_{0}H=4.6$ T while toggling 10-mW, 105-GHz resonant microwave illumination of the sample on and off in 20-s steps. (D) Data with microwaves on, which, compared to the beam off data in (E), suppresses the intensity of excitations from the ground state and increases the intensity of ℏω<0 neutron energy gain excitations from excited states. (**F**) Energy-level scheme for the molecular singlet ground-state magnet with indication of the transition that the 105-GHz microwaves resonate with. Frames (D) to (F) were adapted from (*28*). (**G** and **H**) Inelastic magnetic neutron scattering from spin waves in the 2D square lattice antiferromagnet Rb_2_MnF_4_. (G) Sample in thermal equilibrium at *T* = 3.6 K. (H) Data acquired while the sample is exposed to a 515-nm laser light, which creates a nonthermal magnon population as indicated by the appearance of ℏω<0 neutron energy gain scattering. Frames (G and H) were adapted from (*29*).

Monopole dynamics in spin ice is of great fundamental interest, but it is not accessible to conventional inelastic neutron scattering because the neutron cannot flip Dy or Ho Ising spins and because the characteristic energy (time) scale is too small (long). Figure 4 shows that it is nonetheless possible to probe monopole dynamics through time-resolved magnetic neutron diffraction from a sample driven by a time-dependent applied magnetic field (*25*). We recall that in zero field, there is local order in spin ice but no long-range order and so no magnetic Bragg peaks (Fig. 2). Figure 4 (A and B) shows that a magnetic Bragg peak at (002) is induced by the application of a small field on the spin ice compound Ho_2_Ti_2_O_7_. Periodically toggling the field from on to off in 10-s steps, the experiment provides access to the temperature-dependent timescale for the magnetization and subsequent magnetic relaxation processes. Figure 4C quantitatively shows that the magnetic Bragg intensity is extracted from the diffuse scattering. In the monopole language, the experiment follows the polarization and subsequent relaxation of a thermal population of oppositely charged monopoles. The experiment provided evidence for a cross-over from dipolar to monopolar magnetic relaxation upon cooling (*25*).

Cr_8_F_8_Piv_16_ contains molecular rings of eight Cr^3+^ spin-3/2 degrees of freedom with antiferromagnetic interactions (*26*). It has a singlet ground state and a large number of excited states (Fig. 4F) that can be classified by the total angular momentum and accessed by inelastic magnetic neutron scattering (*27*). Figure 4D shows the low-energy part of the magnetic excitation spectrum as probed by cold neutron inelastic magnetic neutron scattering in a 4.6-T magnetic field at T=2.3(1) K (*28*). A significant change is observed in this spectrum when the sample is exposed for 20 s to 105-GHz microwaves that resonate with the ∣1⟩↔∣2⟩ transition between the first two excited states. The reduced intensity of the 0.8-meV ∣0⟩→∣2⟩ transition indicates a significant depopulation of the singlet ground state, although the measured sample temperature changes by less than 0.1 K. The experiment was able to separately extract time-dependent effective temperatures for each energy level and demonstrate the creation of a nonthermal state through pulsed resonant microwave illumination.

Rb_2_MnF_4_ is a long-range ordered antiferromagnet with a 0.6-meV gap in the magnetic excitation spectrum, and it was recently used to demonstrate time-resolved inelastic neutron scattering at the spallation neutron source (*29*). Figure 4G shows the magnon spectrum for wave vector transfer near the $\left(\frac{1}{2}\frac{1}{2}0\right)$ wave vector and the minimum in the dispersion relation at T=3.6K. A weak intensity is observed for ℏω<0 corresponding to the annihilation of thermally activated magnons. The sample was then exposed to nonresonant laser illumination with 2.4-eV photons. Figure 4F shows that this enhances the (ℏω<0) magnon annihilation part of the cross section, which indicates that the magnon density grows and the spin system is driven out of thermal equilibrium. The data were acquired 51.7 ms after the laser excitation, and the time-dependent annihilation intensity indicates a magnon lifetime in excess of 100 ms that would not be spectroscopically accessible. The experiment shows that the magnon-phonon interactions are ineffective in annihilating the lowest-energy magnon. These early time-resolved neutron scattering experiments are opening an exciting new window into time evolution in interacting spin systems.

## RESONANT X-RAY SCATTERING AS A PROBE OF MAGNETIC ORDER AND COLLECTIVE EXCITATIONS

**Fig. 5.**
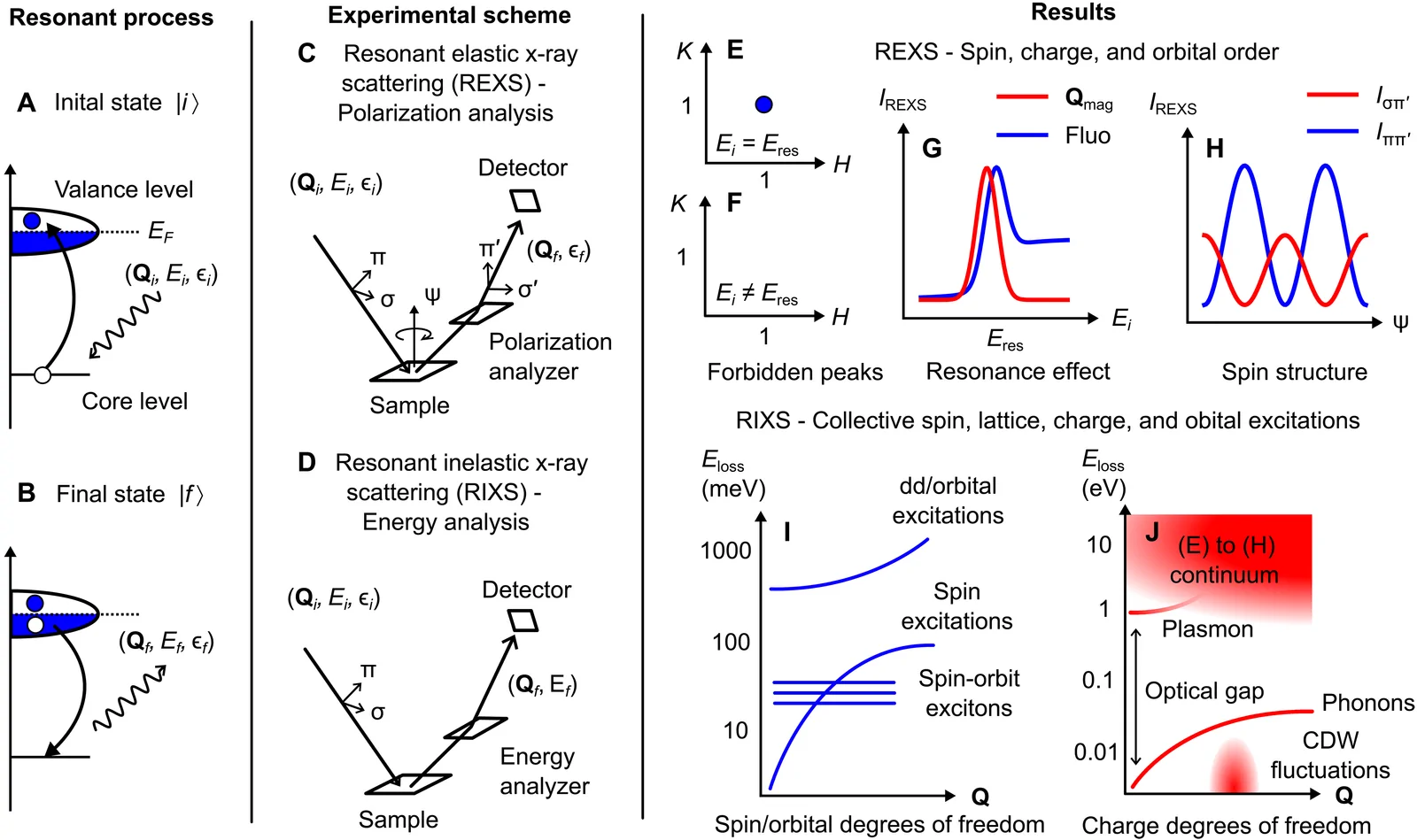
Summary of RXS techniques. (**A** and **B**) Schematic of the initial and final states in the resonant process. (**C** and **D**) Experimental schematic for resonant elastic and inelastic x-ray scattering. Note that although the schematic in (D) is drawn without polarization analysis, polarimetric RIXS setups do exist, although they are not routinely used because of very low count rates. (**E**) Schematic energy dependence of magnetic scattering located at $Q_{\text{mag}}$ and fluorescence. (**F**) Schematic of azimuthal dependence REXS, taken with various linear polarization analysis schemes. (**G** and **H**) Schematic dispersions for spin, orbital, and charge excitations obtained with RIXS. (**I** and **J**) Schematics of various spin and charge excitations occurring at various energy levels.

Resonant x-ray scattering (RXS) is an x-ray scattering measurement in which the incident energy of an x-ray excites a core electron to a valence state, corresponding to an atomic absorption edge contained in the sample (Fig. 5, A and B). The resonant process vastly increases the scattering cross section and gives new information about spin and orbital degrees of freedom. In contrast to x-ray magnetic circular dichroism (XMCD) and x-ray magnetic linear dichroism (XMLD) discussed in later sections, RXS is instead a momentum-resolved scattering process that allows for interrogation of states at finite momentum [e.g., antiferromagnets, charge density waves (CDWs), orbital textures, ...].

When considering the scattering of light from an atom, the leading photon-in-photon-out term is conventional Thomson scattering, where the cross section scales with the square of the Fourier transform of the electron density. Thomson scattering gives us the structure factor *S*(*Q*) (imparting information about density-density correlations) weighted by the electronic form factor (providing information about the spatial extent of the electron), which is measured, for instance, in standard x-ray diffraction measurements. A second photon scattering term, however, is given by the Krammers-Heisenberg formula, and its relevance diverges near a transition energy. This is because photons now have a resonant coupling to a specific electronic excitation process.

This resonant process is a major reason why resonant x-ray techniques are so powerful. The main practical advantage of RXS is the ability to enhance coupling to electronic degrees of freedom beyond trivial charge density, such as their spin and orbital degrees of freedom, and to achieve enhanced contrast of chosen elements in a given material via selection of specific atomic transitions associated with them. There are two types of RXS techniques that we discuss here, which can be categorized into whether the scattered photon has had its energy analyzed. If the scattered photons are not energy analyzed, then they provide an energy integral of x-rays scattering into a particular solid angle with coupled **Q** and energy transfers. This process is typically dominated by elastic, Bragg scattering, and the technique is referred to as resonant elastic x-ray scattering (REXS) (Fig. 5C). If, instead, an analyzer is used to determine the energy of scattered photons, then the inelastic and elastic scattering processes can be isolated, and the technique is called resonant inelastic x-ray scattering (RIXS) (Fig. 5D). While only differing in the experimental scheme by energy analysis, what one can learn from the results can be quite different.

### REXS and RIXS as probes of magnetism

Non-RXS (NRXS) is dominated by the charge degree of freedom, and, while NRXS does couple to magnetism (*30*, *31*), it is an exceedingly weak process such that $\sigma_{\text{mag}}/\sigma_{\text{charge}}∼10^{-6}$. Detecting magnetic order in NRXS typically requires tight resolution matching between the beamline and the sample and mitigation of multiple scattering effects (*32*). In moving beyond this, REXS harnesses resonance effects to amplify the magnetic scattering cross section and resolve static magnetic order. The resonant x-ray cross section was formulated by Hannon *et al.* (*33*), following experimental work by Gibbs *et al.* (*34*), who observed a resonant magnetic enhancement in Ho. The Hannon formalism was later reformulated by Hill and McMorrow (*35*), and this remains a common framework for interpreting magnetic REXS scattering.

The scattering cross section can be expanded in terms of electric dipole-dipole (E1-E1), dipole-quadrupole (E1-E2), and quadrupole-quadrupole (E2-E2) transitions. On resonance, photons excited to an unoccupied state with unpaired moments will increase the magnetic scattering via the polarization and exchange splitting of the band. Formalism for higher-order multipole scattering has also been developed (*36*), and, together, these terms account for the majority of reported magnetic textures. Examples include magnetism in Ho (E1-E1) (*34*), magnetic scattering in hematite (E2-E2) (*37*, *38*), and (E1-E2) scattering in KCr_2_O_4_ (*39*).

Magnetic and charge scattering can have different polarization dependences, where, in the dipole-dipole approximation, charge scattering will not change linear polarization (i.e., $\sigma \to \sigma^{\prime }$ and $\pi \to \pi^{\prime }$) but magnetic scattering can (i.e., $\sigma \to \pi^{\prime }$ and $\pi \to \sigma^{\prime }$). Therefore, magnetic scattering can be further isolated by polarization analysis and control of the incident photon polarization (Fig. 5, E to H). Combining this with measuring the azimuthal dependence of scattering (rotation about a given scattering wave vector), REXS allows for magnetic moment orientations and structures to be directly resolved (*35*). More complete reviews of REXS can be found in (*40*, *41*).

When the energy of the scattered photon is analyzed and photon gains or losses in energy can be resolved, this becomes RIXS. While non-RIXS measures the dynamical charge structure factor S(Q,ω) (the Fourier transform of charge density-density correlations in space and time), RIXS is also capable of accessing spin and orbital degrees of freedom. During the resonant process, the system absorbs a photon, which is excited into an intermediate state having a core hole. The photon then decays to the final state while emitting a photon. This intermediate state is responsible for generating the excitations accessed on resonance (Fig. 5). Here, we will give a brief history of RIXS, and more dedicated reviews can be found in (*42*, *43*).

Following seminal work on NiO (*44*), RIXS emerged as a probe of correlated materials; however, early work was limited by poor energy resolution, e.g., measuring excitations associated with ligand charge transfer in Nd_2_CuO_4_ (*45*). As energy resolution improved, the energy scales of excitations that could be resolved decreased. dd excitations—that is, orbital excitations within d levels—were observed by Ghiringhelli and coworkers (*46*) in various cuprates. These excitations are dipole forbidden but observable with RIXS due to the two dipole transitions associated with absorption and emission. If the dd excitations disperse, then they are known as orbital excitons and can occur due to spin-orbital separation (*47*).

With further improvements in energy resolution, spin excitations were first observed via RIXS in S=1/2 La_2_CuO_4_—first as a nondispersing bimagnon using O–*K* edge RIXS (*48*) and later as a dispersing magnon using Cu–*L* edge RIXS (*49*). The bimagnon is a total ΔS=0 excitation consisting of two entangled spin flips on separate sites, while the magnon is a normal ΔS=1 excitation that neutrons measure (*50*). *L*-edge RIXS is able to probe spin flips as the 2p core hole has finite spin-orbit coupling. Higher-order spin excitations are also observed in higher-spin systems, e.g., in ΔS=2 excitations in *S* = 1 NiO (*51*, *52*). While, historically, the development of RIXS has followed early transition metal edges in the soft x-ray regime, which makes use of multilayer-based optics, high-resolution hard x-ray RIXS, which makes use of crystal optics, is now viable. In the hard x-ray regime, magnons were observed in Sr_2_IrO_4_ using a recently built hard x-ray RIXS spectrometer at the Ir *L* edge (*53*), and, more recently, excitations were probed in the tender x-ray regime in SrRu_2_O_2_ using a tender x-ray RIXS spectrometer at the Ru *L* edge (*54*). A broad array of electronic excitations can be probed via RIXS, ranging from excitations of the charge, lattice, orbital, and lattice degrees of freedom (Fig. 5, I and J).

Today, both REXS and RIXS techniques are complementary tools to neutron scattering, with the abilities to provide full magnetic structural solutions and determine underlying spin Hamiltonians in situations where neutrons are not viable. Because of the higher flux of synchrotron sources, small volumes of scattering can be probed, the dynamics of thin films can be studied, and compounds with neutron absorbing elements (e.g., Gd) can be studied. Advances in optics have pushed the resolving power of RIXS spectrometers to an energy resolution approaching that of neutrons (∼10 meV) (*55*, *56*). Advanced x-ray focusing optics can further allow for tens of nanometers spatial resolution mapping to be performed in targeted x-ray measurements (*57*), and resonant scattering in dark-field resonant x-ray tomography is an emerging frontier for mapping magnetic textures throughout a sample’s volume (*58*–*60*).

### Spin-orbit–entangled states in quantum materials

The ground-state wave functions of magnetic ions in quantum materials are determined by the interplay of multiple energy scales. These include, for instance, the CEF strength, the on-site Coulomb repulsion *U*, spin-orbit coupling strength λ, on-site magnetic exchange or Hunds coupling, and additional electron-lattice interactions that quench orbital degeneracy (e.g., Jahn-Teller distortions). The valence electrons of atoms have differing balances of these energy scales, where, for instance, their oxidation states and atomic numbers bias the relative importance of the CEF and λ, while the spatial extent of the orbital often biases the importance of *U*. In certain regimes, the spin and orbital components of the electronic wave function become inextricably entangled with one another. This is driven fundamentally by λ, and in quantum materials, these spin-orbit–entangled wave functions appear predominantly in two limits defined by the ratio of λ to the CEF energy scale.

The first limit occurs in compounds where λ is much larger than the CEF scale, rendering a direct mixture of *L* and *S* quantum numbers into a total *J* state whose $m_{J}$ degeneracy is then lifted by the weaker perturbation of the CEF. The remnant degeneracy of the spin-orbit–entangled state split by the CEF generates an *S*_eff_ ground state (e.g., $S_{\text{eff}}=1/2$ for twofold degeneracy). This is typically the realm of highly localized 4f electrons in lanthanides. Their ground-state wave functions are complex mixtures of $m_{J}$ states, imparting, in some cases, strong anisotropies into the magnetism of the material. Absent hybridization with conduction electrons, the intrinsic magnetic exchange scales associated with these highly localized states are typically small (<1 meV), and they provide compelling material platforms whose properties can then be tuned with accessible magnetic fields and whose magnetic Hamiltonians can be unambiguously determined. Many compounds with $S_{\text{eff}}=1/2$ wave functions in frustrated lattices are of interest as quantum spin liquid candidates, [e.g., Yb2Ti_2_O_7_ (*61*), YbMgGaO_4_ (*62*), and NaYbO_2_ (*63*)] such as the rare-earth delafossites discussed in the “Resonant X-Ray Scattering as a Probe of Magnetic Order and Collective Excitations” section.

**Fig. 6.**
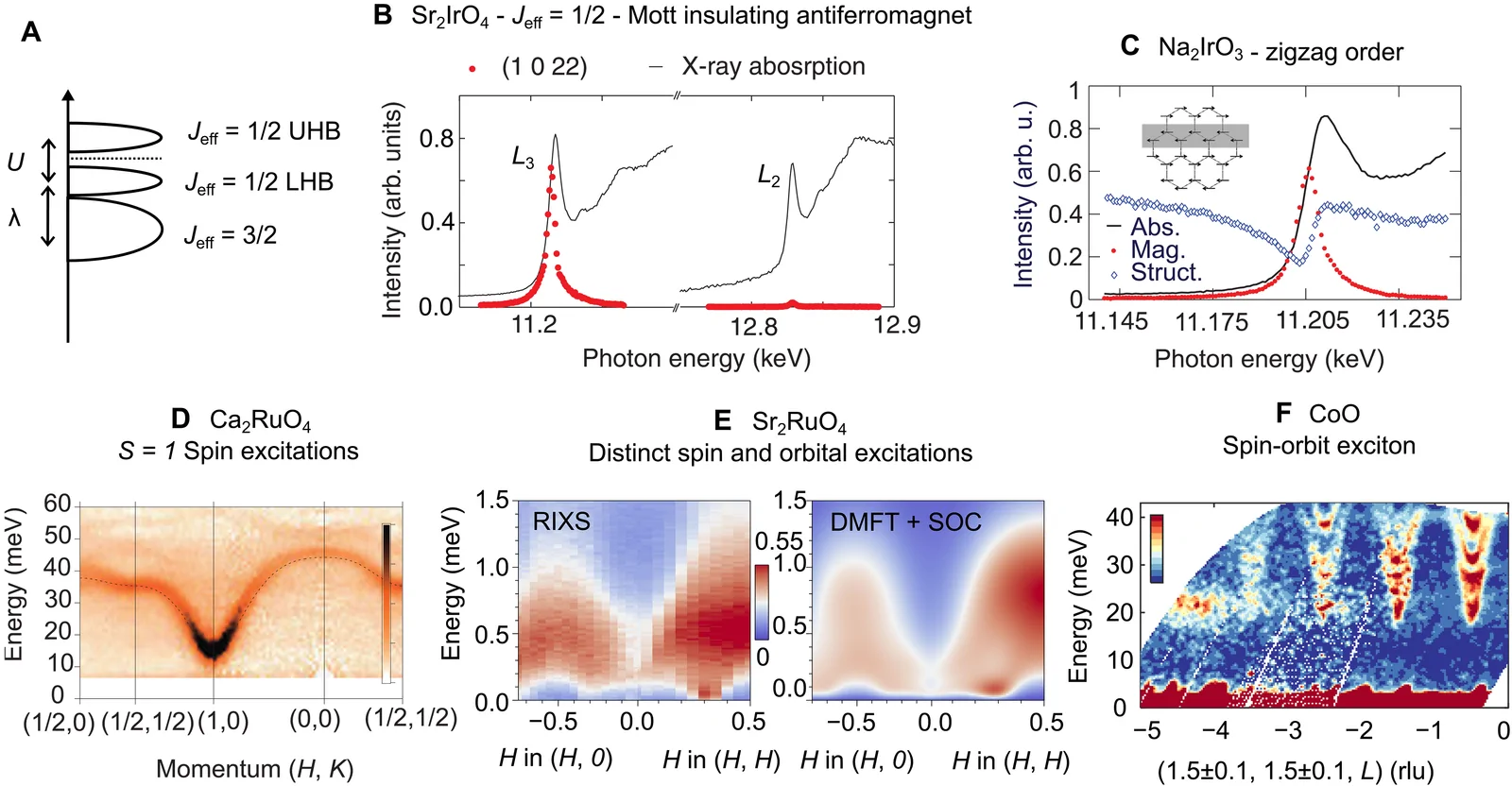
Spin-orbit–assisted phenomena in transition metal oxides probed with neutrons and x-rays. (**A**) Energy-level schematic of a $J_{\text{eff}}=1/2$ Mott insulator. (**B**) Energy dependence of magnetic (1,0,22) Bragg peak in Sr_2_IrO_4_, reproduced from (*68*). (**C**) REXS measurement of zigzag order in Na_2_IrO_3_. Reprinted with permission from (*92*) Copyright (2017) by the American Physical Society. (**D**) Magnetic excitation spectrum of Ca_2_RuO_4_, measured with neutron scattering. Reproduced from (*117*) with permission from SNCSC. (**E**) RIXS excitation spectrum of Sr_2_RuO_4_, reproduced from (*295*), which is licensed under http://creativecommons.org/licenses/by/4.0 CC BY 4.0. No changes were made to the figure. (**F**) Magnetic excitations in CoO measured with neutron scattering. Reprinted with permission from (*128*). Copyright (2019) by the American Physical Society.

Because of their larger energy scales, here, we focus on the second common limit that occurs in compounds where dominant CEF effects lift the orbital degeneracy but fail to fully quench the orbital moment *L*. The remaining λ then mixes the reduced $L_{\text{eff}}$ with *S*, and a $J_{\text{eff}}$ spin-orbit–entangled wave function forms, assuming that the bandwidth is sufficiently narrow to avoid mixing appreciably with other nearby states. Key examples here occur in the 4d and 5d electrons of transition metal oxides with iridates and ruthenates being the most broadly studied platforms. Canonical examples are compounds with Ir^4+^ (5d^5^) ions that are octahedrally coordinated by ligands. The large CEF induces a low-spin state with unquenched orbital degeneracy in the $t_{2g}$ manifold of orbital states, leaving $L_{\text{eff}}=1$. These states are then mixed via λ into $J_{\text{eff}}=1/2$ and $J_{\text{eff}}=3/2$ bands, creating a half-filled $J_{\text{eff}}=1/2$ level and filled $J_{\text{eff}}=3/2$ band (Fig. 6). Interactions here often arise in extended transition metal orbitals, making the exchange coupling larger, excitations more energetic, and more accessible to the energy resolution of RIXS techniques.

#### Spin-orbit–assisted Mott insulators

A longstanding focus in the study of condensed matter is the exploration of emergence and many-body physics in Mott states (1/2-filled bands where *U* opens a gap). For 3d electrons, e.g., Cu 3d^9^ in La_2_CuO_4_, this is typically embodied by compounds with a strong Coulomb repulsion *U* and narrow bandwidth *W*, enough to split an otherwise metallic valence band into upper and lower Hubbard insulating bands. Taking into account the energy scale of charge transfer to the ligands Δ, there exists a spectrum of Mott-Hubbard insulators in the Zaanen-Allen-Swatrtzky scheme (*64*); however. in systems with spin-orbit–entangled wave functions, spin-orbit–driven quenching of orbital degeneracy is also required to create the starting $J_{\text{eff}}=1/2$ half-filled band.

Sr_2_IrO_4_ is a seminal example of such a spin-orbit–assisted Mott insulator (*65*, *66*). It has a low-spin 5d^5^ configuration that gives rise to the $J_{\text{eff}}=1/2$ pseudospin, similar to spin S=1/2 of La_2_CuO_4_. The $J_{\text{eff}}=1/2$ band is narrow enough that the modest *U* of the extended Ir 5d states is enough to split the valence band into an upper and lower Hubbard band (Fig. 6A), creating an insulating antiferromagnetic Mott state that is proposed to superconduct when doped with charge carriers (*67*). REXS techniques played a pivotal role by identifying the antiferromagnetic ground state and using matrix elements of the resonant scattering process as a phase-sensitive tool for confirming the spin-orbit–entangled nature of the moments’ wave function (Fig. 6B) (*68*). Neutron diffraction experiments later confirmed the presence of canted antiferromagnetic order (*69*, *70*); however, the high-absorption cross section and large magnetic bandwidth made inelastic neutron scattering experiments challenging. Instead, RIXS measurements played a pivotal role in determining the spin Hamiltonian of this compound, where the magnetic excitation spectrum is well described with spin wave theory (*53*) and parallels the excitation spectrum of La_2_CuO_4_ (*50*).

Other examples in the same homologous series include the bilayer iridate Sr_3_Ir_2_O_7_, which is also a $J_{\text{eff}}=1/2$ magnetic insulator. It is a G-type antiferromagnet. Its ground state was again resolved by REXS (*71*, *72*) and confirmed with neutron diffraction (*73*). RIXS measurements revealed an unconventional magnetic excitation spectrum with an enormous excitation gap (*74*), which was also modeled as excitations near a dimer instability (*75*, *76*). Later, RIXS studies detected the presence of an extra longitudinal mode and proposed an intrinsic excitonic insulator phase (*77*). There are numerous other examples of spin-orbit Mott states (*78*), and RXS techniques continue to play a key role in exploring their properties and competing phases as their electron filling is tuned (*79*, *80*).

#### Kitaev magnets

In a magnetic system where there are multiple exchange interactions that cannot be simultaneously satisfied, long-range magnetic order can be suppressed, and quantum fluctuations dominate, rendering a ground state of fluctuating spins with no local order parameter or a quantum spin liquid state (*81*). There are a variety of ways to attempt to create these states in families of geometrically frustrated lattices, and one special case for spin-orbit–entangled $J_{\text{eff}}=1/2$ states was proposed for the case of honeycomb lattices. Namely, for $J_{\text{eff}}=1/2$ states built from $t_{2g}$ orbital states, interactions across a honeycomb network were shown to cancel isotropic Heisenberg terms and instead yield bond-directionally dependent interactions that are frustrated at the nearest-neighbor level (*82*). These interactions map to an exactly solvable model for a spin liquid proposed by Kitaev (*83*) and an expansive field grew from studying materials manifestations of these proposals with a recent review found in (*84*).

An early proposal for a material that hosts appreciable Kitaev interactions was Na_2_IrO_3_ (*82*, *85*), where a Kitaev-Heisenberg model was introduced to explore possible ground states. While α-Na_2_IrO_3_ (*86*) and α-La_2_IrO_3_ (*87*) were found to order antiferromagnetically at low temperatures, they nevertheless show signs of appreciable Kitaev-type interactions (*88*). A variety of polymorphs with the necessary ingredients of edge-sharing networks of IrO_6_ octahedra with $J_{\text{eff}}=1/2$ wave functions (*89*) were also explored (*90*, *91*), and, here, we briefly highlight two Kiatev spin liquid candidates and the impact of scattering measurements in understanding their ground states.

REXS (*92*) and neutron scattering experiments (*93*, *94*) observed a “zigzag” antiferromagnetic order in Na_2_IrO_3_ (Fig. 6C); however, crucially, analysis of magnetic diffuse scattering in this material directly resolved the importance of bond directional interactions, a signature of Kitaev physics (*95*). RIXS measurements on Na_2_IrO_3_ characterized the charge gap (*96*, *97*), which, in combination with density functional theory modeling, demonstrated a Mott state where *U* and λ have similar energy scales expected for a spin-orbit Mott state. Further RIXS studies of Na_2_IrO_3_ revealed a magnetic excitation spectrum with a broad continuum (*98*, *99*), and high-resolution RIXS data provided detailed parameterization within a Heisenberg-Kitaev model (*100*). Pushing this further, by performing a topochemical insertion of hydrogen, H_3_LiIrO_6_ can be created, and long-range magnetic order is suppressed, suggestive of a quantum spin liquid state (*101*). A momentum-independent continuum of spin excitations was observed in this regime with RIXS (*102*).

Although not strictly an oxide, a second major avenue of exploring Kitaev interactions in the solid state was through investigations of the compound RuCl_3_. This material is again built of a honeycomb lattice of $J_{\text{eff}}=1/2$ Ru^3+^ moments in a geometry identical to Na_2_IrO_3_. Despite the smaller atomic number of Ru, spin-orbit coupling is still sufficient to create a spin-orbit–entangled $J_{\text{eff}}=1/2$ Mott state (*103*), as verified via RXS (*104*). Neutron diffraction established a low-temperature zigzag antiferromagnetic order that forms below 7 K (*105*); however, inelastic neutron scattering showed that the dynamics associated with this state are reflective of strong Kitaev interactions present in the Hamiltonian (*106*). An in-plane magnetic field can be used to drive RuCl_3_ into a quantum disordered state with continuum dynamics, suggestive of a Kitaev spin liquid state (*107*). Other frustrated 4d^5^ systems with $J_{\text{eff}}=1/2$ ground-state wave functions are also actively being explored, with one recent example in oxides having strong Kitaev interactions reported in NaRuO_2_ (*108*–*110*).

#### Spin and orbital excitations in transition metal oxides

Looking at other transition metal oxides where the spin-orbit coupling is less dominant, there exist numerous compounds with multiple orbitals at the Fermi level and where Hunds coupling acts as a source of correlations (*111*). While a d^5^ system with strong SOC gives a $J_{\text{eff}}=1/2$ ground state, a d^4^ system will give a nonmagnetic $J_{\text{eff}}=0$ ground state (*112*). However, in cases where λ is weaker or comparable to both the magnetic exchange and the crystal field, a complicated energy landscape can emerge. As one such example in 4d transition metals, Ca_2_RuO_4_ is a 4d^4^ magnetic insulator with antiferromagnetic order below $T_{N}\approx 110$ K (*113*) and orbital order near 260 K (*114*). It undergoes a metal-to-insulator transition near 360 K (*115*) and is a candidate for an orbital-selective Mott state (*116*). Both neutron and x-ray scattering techniques have played key roles in understanding its unusual electronic ground state.

INS experiments measured gapped magnons (*117*, *118*) and a longitudinal Higgs-like mode (*117*) in the excitation spectrum of Ca_2_RuO_4_ (Fig. 6D). The observation of a Higgs-like mode suggests proximity to a magnetic instability and a corresponding quantum critical point (QCP) (as will be discussed later). The transverse magnon excitations were modeled with both isotropic (*118*) and anisotropic (*117*) *S* = 1 Heisenberg models affected by spin-orbit–driven anisotropies. RIXS measurements detect the same modes, albeit with poorer energy resolution (*119*), and RIXS data are explained via an atomic multiplet picture, taking into account Hund’s coupling and treating SOC as a perturbation. This picture and proximity to a nonmagnetic $J_{\text{eff}}=0$ (*112*) QCP are consistent with a rich energy landscape and excitonic modes of magnetic order (*120*).

As a 4d^4^ comparator whose interactions favor paramagnetism, Sr_2_RuO_4_ is an unconventional superconductor with $T_{c}\approx 1.5$ K, whose pairing state remains under investigation (*121*). It is isostructural to La_2_CuO_4_, and Sr_2_RuO_4_’s normal state is a correlated nonmagnetic metal (*122*), although incommensurate spin fluctuations associated with Fermi surface nesting and a nearby magnetic instability are observed in scattering measurements (*123*). The role of SOC on the ground state is pronounced, although more subtle than in the iridates. First, SOC is necessary to describe the band structure (*124*, *125*). Second, DFMT taking into account SOC and Hunds coupling can explain the renormalised mass (*126*), and DMFT is needed to describe the excitation spectrum measured with RIXS (*54*), which shows distinct spin and orbital excitations (Fig. 6E).

Looking at even lighter 3d transition metal ions, Co^2+^ often occupies a high spin d^7^ configuration, resulting in a S=3/2 and *L* = 1 state for octahedral crystal fields. The ground state is naively a $J_{\text{eff}}=1/2$ doublet, with higher-energy $J_{\text{eff}}=3/2$ and 5/2 states. This ground state is highly susceptible to a complicated interplay between λ, local structural distortions, and on-site Coulomb *U* terms. Strong *U* often opens a Mott gap, creating $J_{\text{eff}}=1/2$ magnetic insulators with a rich spectrum of low-energy CEF states, magnons, and spin-orbit exciton modes.

A canonical example of this is the rocksalt compound CoO. Some of the first inelastic neutron scattering measurements of CoO (*127*) and subsequent studies (*128*–*130*) revealed a rich, highly structured excitation spectrum (Fig. 6F). The energy levels are understood as spin-orbit–entangled multiplets and their dispersion are captured via a random-phase approximation model originally formulated for spin-orbit–entangled rare-earth systems (*131*). A similar model also applies to the spin-orbit excitons observed in related Co^2+^-based compounds, such as CoTiO_3_ (*132*).

Connecting to the prior subsection on Kitaev interactions, the presence of $J_{\text{eff}}=1/2$ moments in cobalt oxides has also generated proposals of Kitaev interactions within d^7^ cobaltates with a honeycomb magnetic lattice (*133*, *134*). These theoretical proposals were based on the frustrated ground states found in honeycomb compounds such as Na_3_Co_2_SbO_6_ (*135*, *136*) and Na_3_Co_2_TeO_6_ (*135*, *137*). Both compounds have zigzag antiferromagnetic ground states and display spin-orbit excitons determined via scattering measurements (*138*). This is an ongoing area of study where the relevance of terms in extended Heisenberg-Kitaev-Γ and related models is being explored (*138*, *139*).

### Quantum criticality

**Fig. 7.**
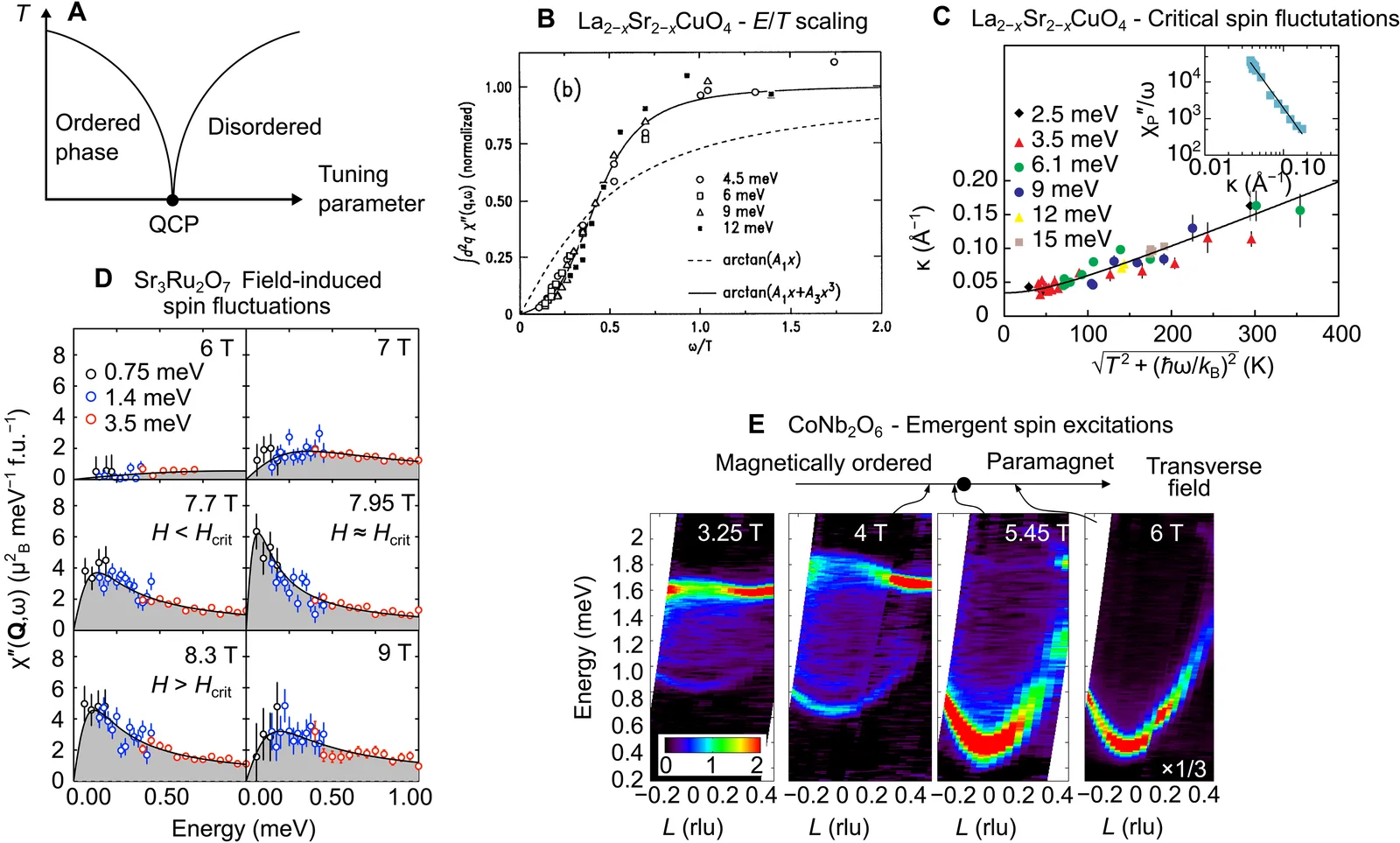
Quantum criticality in transition metal oxides probed by neutron scattering. (**A**) Schematic of a QCP. (**B**) Critical scaling of spin fluctuations in LSCO. Reprinted with permission from (*148*). Copyright (1991) by the American Physical Society. (**C**) Quadrature scaling of the width of spin fluctuations in LSCO. Reproduced from (*296*). (**D**) Dynamic response of spin fluctuations in Sr_3_Ru_2_O_7_ through the critical field. Reproduced from (*174*), which is licensed under http://creativecommons.org/licenses/by/4.0 CC BY 4.0. No changes were made to the figure. (**E**) Magnetic excitation spectrum of CoNb_2_O_6_ though the critical field. Reproduced from (*177*).

When an ordered phase is suppressed to zero temperature by a nonthermal tuning parameter (Fig. 7A), the energy scale of quantum fluctuations becomes comparable to or exceeds that of the thermal fluctuations of the ordered state. This can give rise to a new regime of quantum disordered matter with a plethora of new physics (*140*, *141*). Notably, the excitations born from this instability or QCP are thought to explain unconventional or emergent superconductivity across a number of materials classes such as superconducting copper oxides, heavy fermion compounds, iron pnictides/halcogenides, and Bechgaard salts (*142*).

The quantum critical fluctuations associated with a nearby QCP affect the response of a material over an extended thermal regime—in the case of a suppressed magnetic order parameter, these extend over a thermal regime on the scale of the magnetic exchange strength. Because of these fluctuations, in electron transport, a linear-in-temperature resistivity often appears as a “fan” above the critical point, and an enhancement in the Sommerfeld coefficient γ (often reflective of an increase in the electron’s effective mass) appears. These regimes of strong quantum critical fluctuations can also be mapped in x-ray and neutron scattering measurements. This occurs because of the scale invariance associated with the QCP, and critical scaling occurs in the magnetic excitation spectrum of a material. Spectroscopically, the magnetic spectral weight of the system will scale as a function of E/T as beautifully illustrated by early studies of CeCu_6−*x*_Au*_x_* (*143*). Here, *E* is the energy scale of the fluctuations, *T* is the temperature, and, in certain cases, emergent collective modes may also appear (*144*). These spectroscopic signatures are commonly sampled via scattering techniques and are key indicators of quantum criticality across the phase diagrams of many magnetic materials.

At low-energy scales, scattering experiments are also susceptible to thermal and quantum fluctuations. Thermal fluctuations generally manifest in two ways in a scattering experiment. This is attenuation of elastically scattered photons/neutrons by thermal motion, and thermal repopulation of inelastically scattered photons/neutrons. These are well known and described by the Debye-Waller factor and the Bose factor, respectively. These effects are more pronounced at high temperatures and get greatly reduced when cooling down.

#### Quantum critical scaling in high-temperature superconductors

Quantum criticality is prominently featured in the high-temperature cuprate superconductors, which are obtained by either hole or electron doping a Mott insulating antiferromagnetic parent state (e.g., La_2_CuO_4_). Upon hole (*p*) doping, a rich phase diagram is revealed with various competing phases, and an unconventional superconducting state appears whose mechanism is not yet fully understood (*145*). As they become superconductors, cuprates also exhibit many signatures of quantum criticality. For instance, as a function of hole doping, a linear in temperature resistivity is observed around optimal *p* = 0.16 doping, associated with a “Planckian scattering rate,” where the quasiparticle scattering rate approaches the thermal limit $\Gamma ∼k_{B}T/\hbar$ (*146*). A quasiparticle mass enhancement is also observed around doping *p* = 0.19 (*147*). All this suggests the presence of a putative critical point near p∼0.19 (*145*, *147*), although the ordered antiferromagnetic states of cuprates are rapidly suppressed with carrier doping (p≈0.02).

The low-energy magnetic response or the dynamic spin susceptibility χ″(**Q**,E) (directly related to the magnetic structure factor that neutron scattering measures via the fluctuation-dissipation theorem) is seen to obey E/T scaling, first observed in LSCO and LBCO (Fig. 7, B and C) (*148*, *149*). Recent work shows that these quantum critical fluctuations are present across the phase diagram (*150*) and are able to account for an enhancement in the Sommerfeld coefficient via a spin fluctuation model of heat capacity (*150*). The scaling behavior of the spin fluctuations in the overdoped regime agrees with a quantum Griffiths model, where the QCP is smeared out by disorder (*151*). Cuprates that are instead electron doped similarly show E/T scaling near their antiferromagnetic QCPs (*152*).

In addition to spin fluctuations, fluctuations associated with CDW order are detectable with RIXS in cuprates (*153*) due to recent improvements in both x-ray flux and energy resolution. These CDW fluctuations are observed in a number of variants and show signs in linear in temperature in their energy widths (*154*–*156*), although strict criteria of a CDW critical point, that is, the disappearance of the static CDW and a diverging correlation length, are not observed as a function of doping (*157*). Exploring the interplay of these fluctuations and those from the magnetic degree of freedom and their impact on the superconducting order parameter is an ongoing area of study.

It is worth noting that other classes of nonoxide high-temperature superconductors such as iron pnictides and chalcogenides also feature superconductivity in close proximity to an electronic QCP (*158*). Scattering measurements mapping quantum critical scaling in select variants have been performed (*159*–*161*), albeit not as exhaustively as those in their cuprate counterparts. Last, while they are often intermetallics, the critical E/T scaling generically observed near antiferromagnetic QCPs can also be found in systems with local criticality (*162*), for instance, due to a competition between RKKY and Kondo interactions. Sc_1–*x*_U*_x_*Pd_3_ is one example of this (*163*), as well as UCu_5–*x*_Pd*_x_* (*164*), and similar scaling is found in Kondo semimetals such as CeRu_4_Sn_6_ (*165*).

#### Emergent order and excitations near QCPs

There also exist a host of other compounds with magnetic order that can be driven toward a QCP or magnets with unusual excitations that arise because of the presence of quantum critical fluctuations. As one example, Sr_3_Ru_2_O_7_ is a bilayer member of the Ruddleston-popper series hosting superconducting Sr_2_RuO_4_. Sr_3_Ru_2_O_7_ is a metamagnetic metal—that is, a magnetic phase appears with applied field (*166*). Signs of quantum criticality appear associated with the metamagnetic phase in transport measurements (*167*), and thermodynamic measurements across the critical point show a release of entropy associated with a critical point as well as a Tlog(1/T) divergence of the heat capacity (*168*). Anisotropies in the magnetotransport anomalies associated with the metamagnetic transition generated suggestions of an electronic nematic fluid (*169*).

The origins of the QCP were originally unclear with proposals linked to the metamagnetic phase or with strong Fermi surface fluctuations (*170*). Neutron scattering measurements revealed the presence of field-induced spin fluctuations (Fig. 7D) (*171*, *172*), and with low enough temperatures, the observation of a spin density wave (SDW) state (*173*). The SDW peaks appear coincident with the metamagnetic phase, and its field orientation dependence maps to that of the nematic phase when measured via transport (*173*). The magnetic response of the SDW also sharpens around the critical field in a nearly singular way, as the field is swept through the critical field (*174*), demonstrating that the two features are fundamentally linked.

As an additional example, quantum critical phenomena are often observed in low-dimensional magnetic systems. For instance, E/T scaling is observed in the quasi-1D S=1/2 spin-chain compound KCuF_3_ (*175*) and in the quasi-2D quantum disordered antiferromagnet ZnCu_3_(OH)_6_Cl_2_ (*176*). Here, magnetic order is often tunable via a continuous magnetic field or pressure, and, in the 1D case, a famous example is applying a field transverse to the chain direction of an Ising spin chain to suppress long-range order and induce a so-called quantum paramagnetic phase. A material manifestation of this is the transverse-field Ising model in CoNb_2_O_6_. which forms zigzag chains of $S_{\text{eff}}=1/2$ Co^2+^ with dominant Ising exchange. Emergent excitations were observed by neutron scattering (Fig. 7E) (*177*), where a continuum of spinons (fractionalized S=1/2 quasiparticles) corresponding to the 1D ordered phase is suppressed when a magnetic field is applied. The continuum condenses into bound states, and the emergent bound states, corresponding to an emergent “*E*8” symmetry, soften as the QCP is approached.

Phase (Goldstone) and amplitude (Higgs) modes of fluctuations of a magnetic order parameter may be measured via inelastic scattering techniques. Amplitude modes in magnets are prone to decay into Goldstone modes, making their detection difficult. In proximity to a QCP, however, these amplitude modes may be stabilized. For instance, an amplitude mode sharpens and softens in the TlCuCl_3_ quantum dimer system near its QCP (*178*), and a longitudinal mode appears in the spinon spectrum of quasi-1D KCuF_3_ (*179*). As discussed earlier, a Higgs-like longitudinal mode is also stabilized in Ca_2_RuO_4_ near a $J_{\text{eff}}=0$ QCP (*117*).

#### RIXS on quantum systems

Entanglement among spins is an important concept underpinning quantum many-body systems. While quantifying entanglement is a fundamental concept in quantum metrology, it has also recently found its place in modern condensed matter experiments. The quantum Fisher information (QFI) is a metric for the lower bound of multipartite entanglement. While trivial for a two-level system, it becomes highly nontrivial in a solid-state system with an extensive number potentially entangled particles. The QFI for entangled spins can be obtained as a weighted integral of the dynamical susceptibility obtained by neutron scattering (*180*). It has found many applications in magnetic systems, such as QSL candidates, spin chains, and quantum critical systems. (*181*).

RIXS is sensitive not only to spin but also to orbital and charge degrees of freedom. This makes extracting dynamical susceptibilities less straightforward than neutrons, such that a direct integral introduced in (*180*) is not applicable. There have been several recent works aiming to extract the QFI from RIXS. One involves approximating the dynamical spin susceptibility in the context of a time-resolved study (*182*). Another involves extracting the orbital entanglement by comparing experimental results on the dimer system Ba_3_CeIr_2_O_9_ to RIXS cluster calculations (*183*), and a more recent work involves aim to extract the entanglement of the combined spin-orbital degree of freedom from RIXS spectra (*184*). While the previous works concern the spin and orbital degree of freedom, this has also been applied for NRXS (*185*), which strictly measures the charge degree of freedom.

Another quantum manifestation of RIXS is RIXS interferometry. That is, planes of atoms can serve as a local Young’s double slit experiment in the RIXS process, where the diffraction slits arise from within the crystal lattice. Such an effect was predicted in molecules theoretically in the 1990s (*186*, *187*) and observed experimentally in the dimer system Ba_3_CeIr_2_O_9_, which is made possible because of the two inequivalent sites associated with Ir dimers (*188*). The idea was taken further and applied to pyrochlore Nd_2_Ir_2_O_7_, which forms two inequivalent Ir tetrahedra due to its all-in-all-out magnetic structure. The two sites give an interference-driven fringe pattern, and an entanglement spectrum was extracted that points toward a hidden symmetry breaking in the material (*189*).

## MAGNETIC X-RAY SPECTROMICROSCOPIES TO INVESTIGATE STATIC AND DYNAMIC PROPERTIES OF SPIN TEXTURES

### X-rays—From by-products to x-ray lasers

Investigations of magnetic materials using x-rays did not start until the mid-1980s when it was lastly recognized that the unique properties of synchrotron radiation (SR) created at electron storage rings, specifically their polarization, intensity, energy spectrum, and collimated direction at large scale electron accelerators are highly valuable providing distinct insight into underlying spin textures in scientifically interesting and technologically relevant magnetic systems and structures. Until then, SR was seen as an unwanted by-product for high-energy and elementary-particle physics, which pursued the development of accelerators primarily by the demands for higher electron energies, thus creating larger particle masses but suffering from the fourth power loss of energy. This loss was known since 1897 when J. Larmor and A.-M. Liénard used classical electromagnetic theory to show that the radiated power of a relativistic particle undergoing centripetal acceleration in a circular trajectory scales with ${\left(E/{\mathrm{mc}}^{2}\right)}^{4}/R^{2}$, where *E* is the particle energy, *m* is the rest mass, and *R* is the radius of the trajectory.

X-rays were found in 1895 by Röntgen (*190*) while investigating cathode rays using a vacuum tube. J. J. Thomson’s discovery of the electron in 1897, followed by the postulation of the spin of the electron and its experimental validation in 1925 by W. Pauli, and S. Goudsmit and G. Uhlenbeck, respectively, laid the foundations for today’s approach to understanding magnetism.

Around 1945, Schwinger (*191*) calculated the detailed frequency spectrum of SR and predicted that the radiation would be highly polarized, with the electric field vector lying primarily in the plane of the electron’s orbit. D. Ivanenko, I. Sokolov, A. Pomeranchuk, and others derived asymptotic formulas for the spectral and angular distribution of the radiation, including its polarization components. The experimental exploitation of SR or “Schwinger radiation” has since been intimately connected with progress in accelerator technology. Although the first and second generation of SR sources were solely parasitic users of those high-energy storage rings, this changed completely with the third generation of SR facilities in the 1990s, where the primary goal was to increase the brightness and create and control x-ray polarization solely for the purpose of serving advanced x-ray spectroscopy, scattering, and notably microscopy experiments. Third-generation storage rings are characterized by dedicated insertion devices, such a undulators and wigglers, where the electrons were forced on undulating trajectories, thus optimizing brightness. It is worth noting that in addition to large scale x-ray facilities, there are laboratory-based developments using high–harmonic generation laser systems aiming to reach into the soft x-ray regime, which have also been used for studies of magnetism, albeit limited by the much lower photon flux compared to large-scale SR facilities. With the advent of the fourth generation of large-scale SR facilities, notably diffraction-limited storage rings and x-ray free electron lasers (XFELs) at linear accelerators, the goal today is to push science with x-rays to ultrashort (femtosecond) timescales, full longitudinal and transversal coherence, and at orders of magnitude higher peak intensities than with today’s sources. This opens a path to capabilities that will enable similar transformational scientific achievements as have occurred after the development of lasers with optical wavelengths and energies but now in the x-ray regime with subatomic wavelengths and associated energy levels. Unprecedented insights into fundamentals of chemistry and materials with applications to biology, earth science, quantum phenomena, and technologies are within reach now. The only requirement to use those facilities for studies of magnetic materials is the polarization of the x-rays as the key parameter.

### X-rays and magnetism

Two foundational magneto-optical phenomena govern the interaction of light with magnetic materials. The Faraday effect, which was first observed by Faraday in 1845 (*192*), manifests as a rotation of the transmitted light’s polarization plane, with the magnitude of rotation proportional to the projection of the magnetic field onto the direction of light propagation. The Kerr effect, which was reported in 1877 by Kerr (*193*), by contrast, operates in reflection geometry, modifying both the polarization state and the intensity of light reflected from a magnetized surface. Both effects continue to serve as indispensable experimental probes in the characterization of magnetic materials.

At x-ray energies, the coupling between electromagnetic radiation and magnetic order is encapsulated by x-ray dichroism phenomena (*194*–*196*). XMCD arises from the differential absorption or scattering of left- and right-circularly polarized x-rays in magnetically ordered materials, with its magnitude scaling with the projection of the local magnetic moment onto the photon propagation direction. XMLD, by contrast, probes the anisotropy of the absorption cross section with respect to the orientation of linear x-ray polarization relative to the magnetic moment axis and scales with the absolute value of the magnetization. These two complementary effects serve distinct roles in the investigation of magnetic order: XMCD is particularly suited to ferro- and ferrimagnetic systems, while XMLD provides access to antiferromagnetic order. The emergence of altermagnetism—a magnetic phase exhibiting hallmarks of both ferromagnetic and antiferromagnetic behavior—has motivated combined XMCD and XMLD studies, which have proven effective in characterizing this novel class of magnetic materials (*197*).

**Fig. 8.**
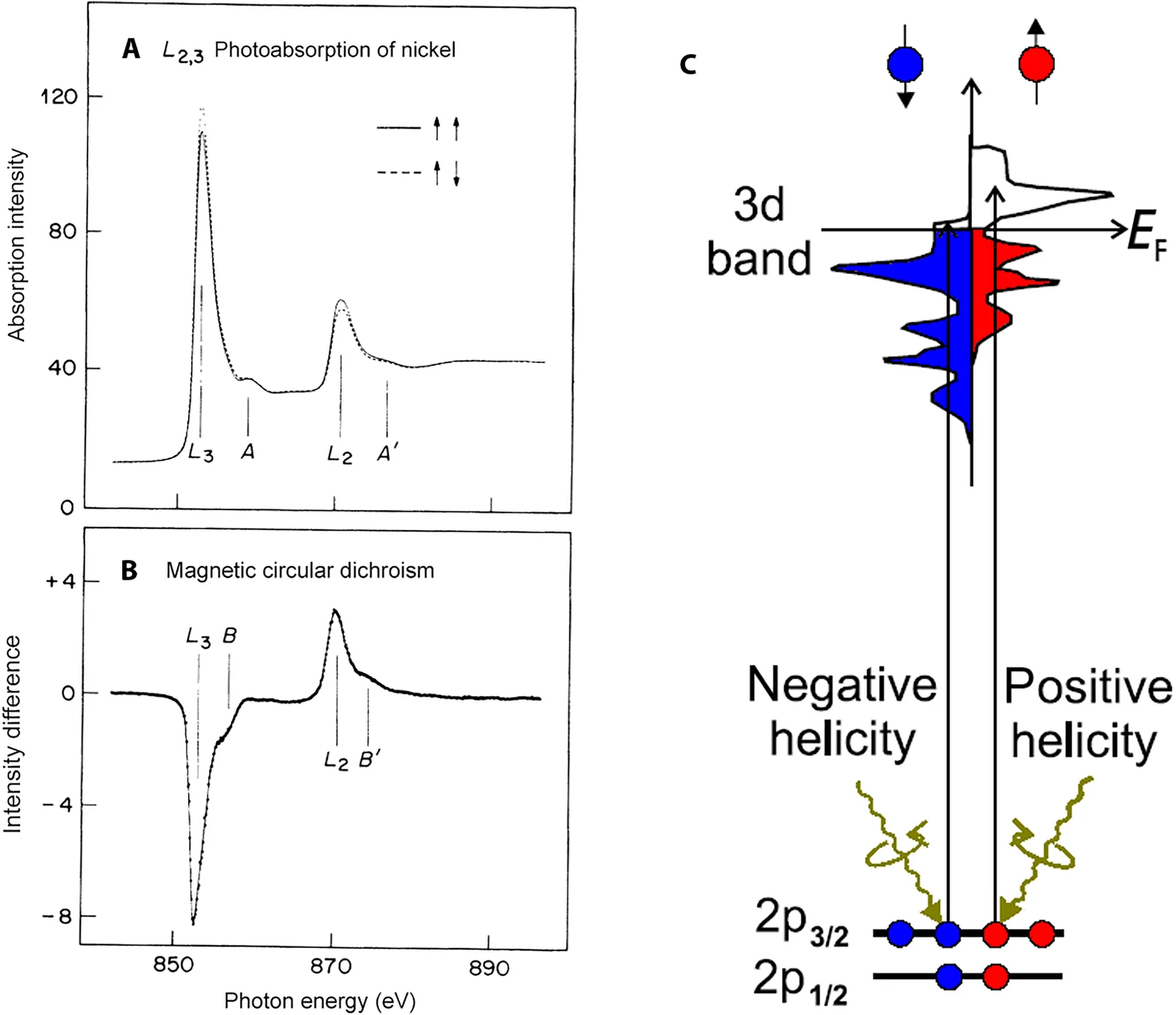
XMCD at $L_{3/2}$ edges. (**A**) First experimental demonstration of x-ray absorption and (**B**) XMCD at the $L_{3/2}$ edges in Ni metal (*202*). Reprinted with permission from (*202*). Copyright (1990) by the American Physical Society. (**C**) Schematics of the XMCD process (*194*).

The microscopic origin of x-ray dichroism is fundamentally different from that of conventional magneto-optical effects. While the latter arise from transitions between extended band states, x-ray dichroism is rooted in resonant core-level spectroscopy: When the x-ray photon energy is matched to the element-specific binding energy of an inner-shell electron, such as the 2p_3/2_ or 2p_1/2_ electrons at the $L_{3}$ or $L_{2}$ edges—distinguished by their total angular momentum $\vec{j}=\vec{l}\pm \vec{s}$ – a dipole-allowed transition (Δl=1) promotes the electron from the p core level into an unoccupied d state at the Fermi energy. The key physical mechanism is the transfer of angular momentum from the circularly polarized photon to the photoelectron via spin-orbit coupling, which renders the excited electron spin-polarized. This spin-polarized electron then probes the exchange split density of states of the FM, experiencing different absorption for the majority and minority spin bands as governed by Fermi’s golden rule. The opposite spin-orbit character of the p_3/2_ (j=l+s) and p_1/2_ (j=l−s) initial states is the physical origin of the sign reversal of the XMCD signal observed between the $L_{3}$ and $L_{2}$ edges, a defining and experimentally well-established signature of the effect (Fig. 8A).

From a spectroscopic standpoint, XMLD effects serve as a directional probe of valence hole occupancy, enabling the determination of local atomic symmetries and the orientation of magnetic spin axes. Their magnitude is generally more modest than that of XMCD, which can reach asymmetries of 20 to 30% in 3d transition metals, although multiplet splitting can substantially enhance XMLD signals in certain systems.

The chronological development of x-ray dichroism as a field unfolded in stages. The theoretical groundwork was established in 1985, when Thole *et al.* (*198*) predicted the existence of strong XMLD effects in rare-earth systems—a prediction rapidly validated through experiment by van der Laan *et al.* (*199*). The complementary effect, XMCD, was first observed experimentally in 1987 by Schütz *et al.* (*200*), who detected subtle differences in the absorption of circularly polarized hard x-rays at the *K* edge of iron. The minute size of the observed effect was a direct consequence of the *s*-symmetry initial state at the *K* edge, which lacks the LS coupling necessary to generate a large dichroic asymmetry. Progress continued with the discovery of more pronounced XMCD signals—on the order of a few percent—at the *L* edges of 4f rare earth and 5d elements in the hard x-ray regime. Systematic exploration of the full 4f lanthanide series through XMCD measurements (*201*) began to foreshadow the transformative impact that this technique would ultimately have on the quantitative characterization of magnetic materials.

The year 1990 marked a turning point for XMCD spectroscopy, when Chen *et al.* (*202*) observed dichroic effects of 20% at the $L_{3,2}$ edges of Ni in the soft x-ray regime (Fig. 8B), igniting widespread research interest. The soft x-ray penetration depth proved ideally suited to the thickness scales of magnetic thin films and multilayers relevant to spintronics and nanomagnetism (*203*). This breakthrough was swiftly followed by theoretical developments of Thole *et al.* in 1992 (*204*) and Carra *et al.* in 1993 (*205*), whose magneto-optical sum rules enabled experimentalists to extract quantitative, element-specific spin and orbital moments directly from XMCD spectra, cementing the role of x-ray spectroscopy as a cornerstone technique in magnetic materials research.

Magnetic x-ray spectroscopy has since become an indispensable tool to characterize magnetic materials. A broad portfolio of specialized measurement platforms has been developed and deployed at synchrotron facilities worldwide, providing experimentalists with sample environments that combine ultralow temperatures extending into the sub-kelvin range (*206*) with high pulsed magnetic fields of up to 40 T, the latter capable of being directed along any crystallographic or magnetic axis of the specimen under study (*207*, *208*).

### Magnetic imaging with polarized x-rays

**Fig. 9.**
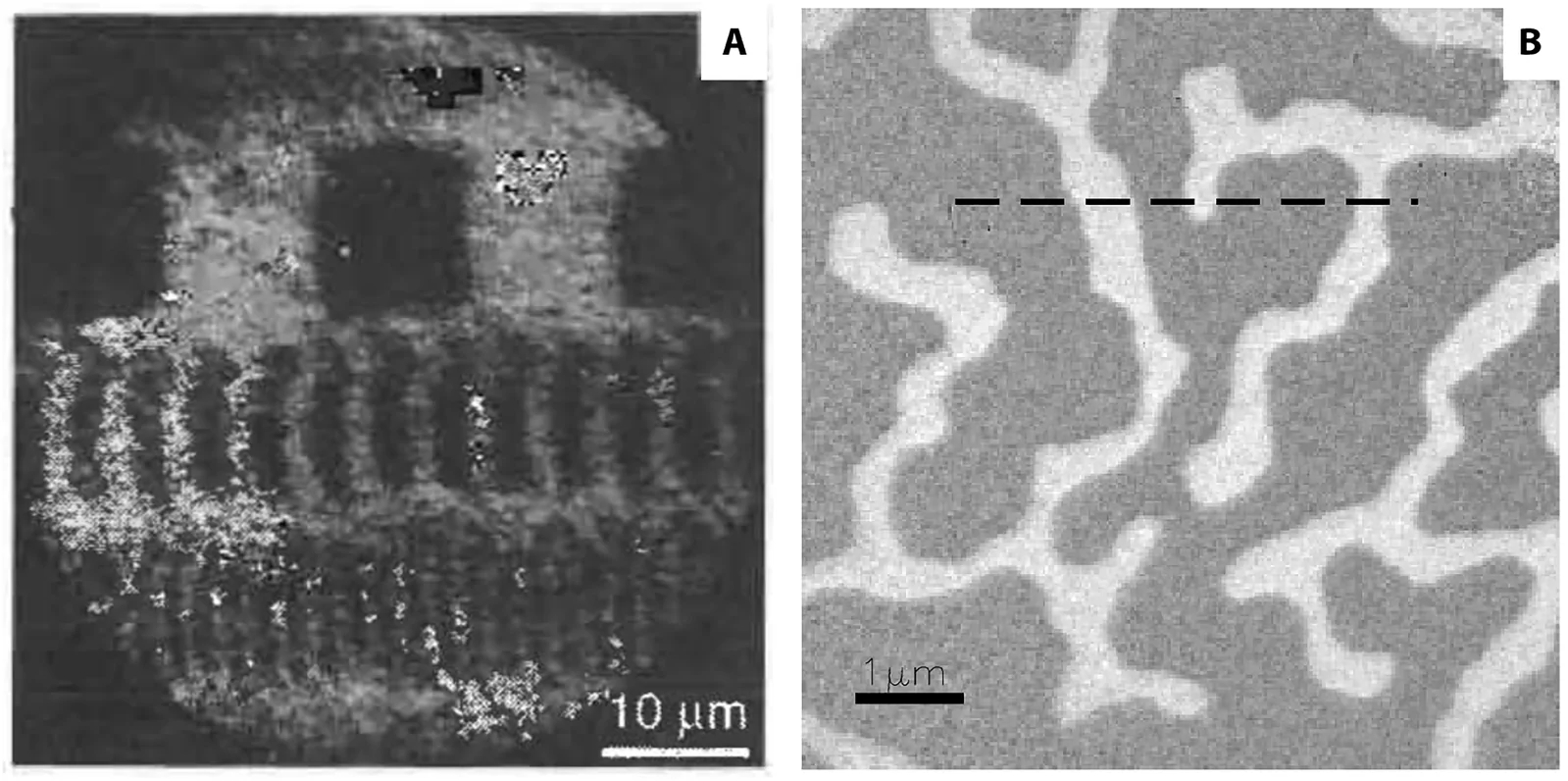
Magnetic soft x-ray microscopy. Images of nanoscale magnetic domains obtained with (**A**) X-PEEM [reproduced from Stöhr *et al.* (*209*), *Science* (1993), AAAS] and (**B**) MTXM. Reproduced with permission from Springer Nature (*210*).

The next milestone was to consider XMCD and XMLD as a distinct magnetic contrast mechanism in magnetic microscopies with the goal of ultimately determining spin textures at nanoscale dimensions. Two pioneering experiments in the early-to-mid 1990s laid the foundation for magnetic x-ray microscopy as we know it today. The first came in 1993, when J. Stöhr *et al.* (*209*) harnessed the power of circularly polarized x-rays at the Advanced Light Source in Berkeley to produce the very first magnetic x-ray image, capturing micrometer-sized magnetic domains on a recording disk using an x-ray photoemission electron microscopy (X-PEEM) instrument and demonstrating that x-rays could serve as a uniquely sensitive and element-specific probe of magnetic domain structure (Fig. 9A). The second milestone followed in 1996, when Fischer *et al.* (*210*) took a different approach at BESSY I in Berlin, building a full-field soft x-ray transmission microscope equipped with Fresnel zone plate optics and using XMCD as a magnetic contrast mechanism to image the domain patterns of a 59-nm-thick GdFe film at a remarkable spatial resolution of 60 nm (Fig. 9B). Together, these two experiments defined the two primary paradigms of magnetic x-ray microscopy, each with its own strengths: X-PEEM’s photon-in/electron-out detection scheme offers surface sensitivity but is fundamentally constrained by the incompatibility of electron optics with strong applied magnetic fields, while magnetic full-field soft x-ray transmission microscopy (MTXM)’s photon-in/photon-out geometry circumvents this limitation entirely, opening the door to nanoscale imaging of magnetic spin textures as they evolve under applied fields across a complete hysteresis cycle (*211*).

Two particularly impactful applications of X-PEEM illustrate the breadth of magnetic phenomena accessible with this technique. In 2000, Nolting *et al.* (*212*) demonstrated the power of XMLD-contrast X-PEEM by directly resolving the microscopic spin alignment between the ferromagnetic and antiferromagnetic sublattices in an exchange bias heterostructure—an observation that provided direct experimental evidence for the interfacial spin coupling mechanism responsible for the exchange bias effect. Subsequently, X-PEEM has been extensively applied to the study of artificial spin ice (ASI) systems, in which arrays of lithographically patterned single-domain magnetic islands are arranged on geometrically frustrated lattices. In a seminal 2011 study, Mengotti *et al.* (*213*) used X-PEEM to image an ASI Kagome lattice and directly identified the signatures of emergent magnetic monopole excitations and their associated topological Dirac strings, providing real-space experimental confirmation of theoretical predictions regarding the nature of magnetic frustration and fractionalization in these engineered systems.

Today, a large suite of x-ray microscopy techniques are available at SR facilities around the world (*214*). Among the real-space x-ray imaging, modalities currently used for magnetic investigations, three techniques occupy a central role: X-PEEM, MTXM, and scanning transmission x-ray microscopy (STXM). In the STXM approach, diffractive focusing of the incident x-ray beam by a Fresnel zone plate produces a diffraction-limited illumination spot at the sample plane. This focused spot is then systematically translated across the region of interest in a raster pattern, with the spatially integrated transmitted photon intensity recorded at each scan position by a point detector—most commonly an avalanche photodiode—to build up a 2D image of the sample. Recent x-ray microscopy developments, specifically those that are pushing the limits for magnetic x-ray imaging, are focusing on reciprocal space techniques and will benefit from the increased coherence at next-generation lightsources. Among those novel techniques that have been extensively used for magnetic imaging are coherent diffractive imaging (*215*), x-ray ptychography (*216*, *217*), and x-ray laminography (*218*) that often leverage STXM setups, whereas x-ray holography (*219*) as a full-field technique has shown its potential for single shot (*220*) and multicolor magnetic imaging (*221*) particularly at XFELs toward studies of femtosecond spin dynamics.

### Probing the limits with magnetic x-ray spectromicroscopy

The strong dichroism effects, specifically in the soft x-ray regime, provide high sensitivity to the detection of properties in magnetic materials. For magnetic x-ray spectroscopy, this relates to the ability to measure spin and orbital moments with high precision, down to fractions of a Bohr magneton. Among the most challenging x-ray microscopy measurements so far in terms of signal-to-noise ratio were advanced STXM experiments capable of detecting spin currents (*222*), recording the switching in nanospin torque oscillators (*223*), observing solitons in magnetic nanocontacts (*224*), and imaging spin waves emitted in permalloy nanostructures (*225*), or PEEM studies on highly dispersed single magnetic nanocrystals (*226*).

### Time-resolved magnetic x-ray microscopy

A new dimension for x-ray investigations of spin textures was entered in the early 2000s by harnessing the inherent pulsed temporal structure of synchrotron facilities to image fast magnetization dynamics with time-resolved magnetic x-ray microscopies. Since each electron bunch circulating in a storage ring at 100 s of megahertz frequencies emits only a few photons every time it passes by, pump-probe modalities were used to accumulate a sufficient photon count needed to record a single image. Varying the delay time between the electronic, magnetic, or optical pump and probing x-ray pulse, a sequence of time-resolved static images could be obtained from which the fully repeatable components of the spin dynamics were determined.

The first pioneering time-resolved magnetic x-ray microscopy studies focused on the vortex dynamics in permalloy nanostructures (*227*), where the inherent magnetization timescales and reliable resetting of the spin texture after each pump pulse were warranted. Switching phenomena of the vortex core (*228*) and its circularity/chirality (*229*) were investigated with those approaches in great detail. The topic of ultrafast spin dynamics, e.g., all-optical switching (AOS) of spins using a femtosecond short optical laser pulse will be discussed in the “Femtomagnetism and subpicosecond AOS of spins” section.

### Topological spin textures

With the growing scientific and technology interest in topological spin textures, such as magnetic skyrmions (*230*–*232*) and their potential as building blocks for ultrasmall and ultrafast low-power electronics, understanding the static properties and dynamic behavior of skyrmion became a focus of investigations with the suite of available magnetic x-ray microscopies. The scientific significance of 2016 for the skyrmion community can hardly be overstated and is deeply rooted in magnetic x-ray microscopy. A trio of landmark studies—by Woo *et al.* (*233*), Moreau-Luchaire *et al.* (*234*), and Boulle *et al.* (*235*)—harnessing the magnetic contrast of MTXM and STXM, demonstrated that magnetic skyrmions could be stabilized at room temperature within multilayered thin-film systems, removing one of the most significant obstacles to their practical use and revealing the material parameters that govern their stability and mobility in racetrack device geometries. The dynamic counterpart to these static observations exploited the time-resolved capabilities of magnetic STXM to directly capture the motion of current-driven skyrmions in real space and time, uncovering the skyrmion Hall effect—a topologically mandated deflection of skyrmion trajectories transverse to the applied current—with profound implications for the design of skyrmion-based spintronic devices. Using time-resolved magnetic x-ray holography Buettner *et al.* (*236*) determined the inertia of skyrmions. Magnetic x-ray laminography resolved the internal spin textures of a single skyrmion in 3D (*237*), and, recently, a combination of MTXM and X-PEEM pushed topological spin textures to a new dimension by validating the stabilization of a magnetic Hopfion in a magnetic multilayer system (*238*).

### 3D nanomagnetism—The need for magnetic nanotomography

**Fig. 10.**
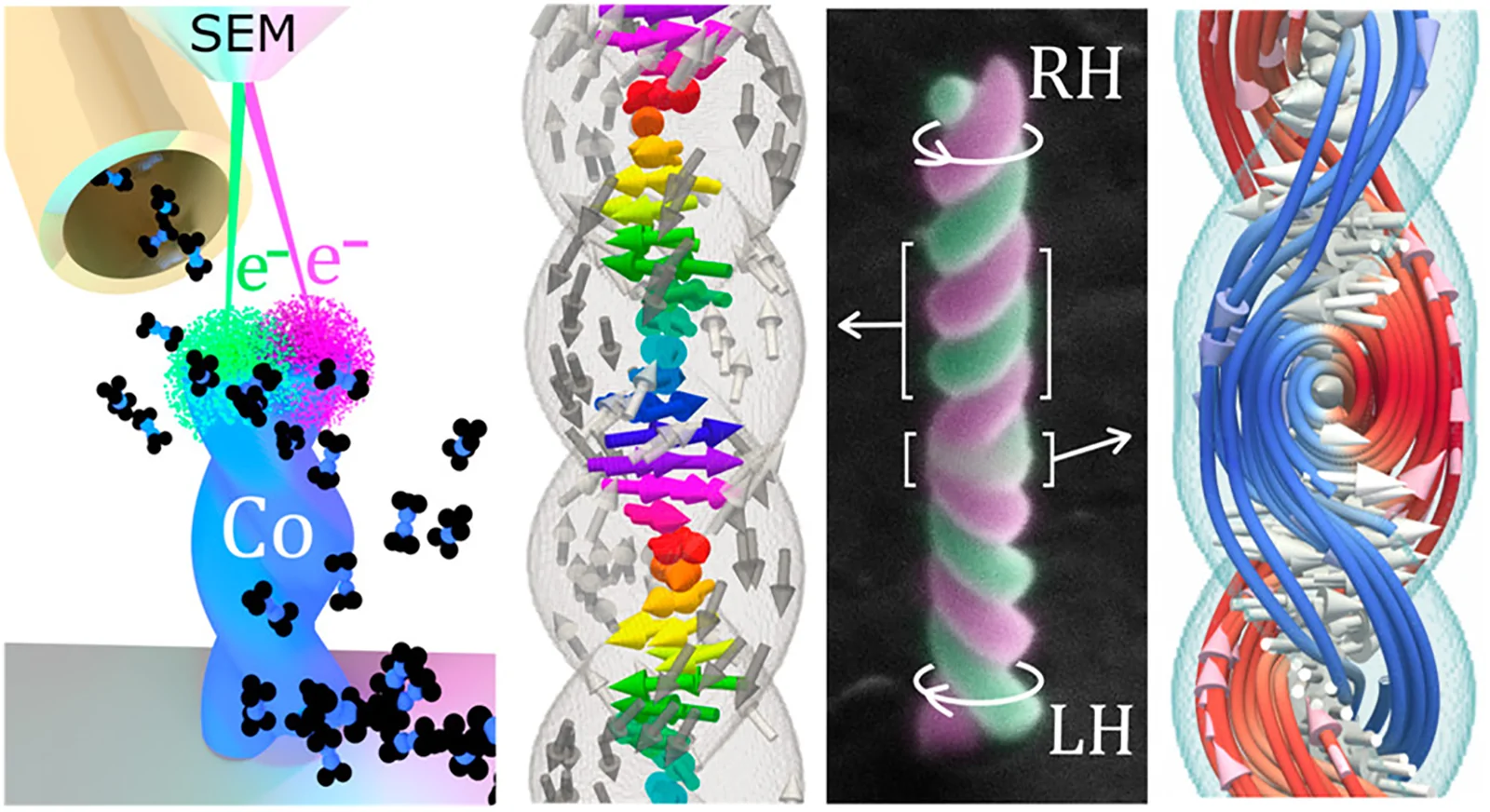
3D spin textures in a twisted magnetic nanowire. Left: Schematics of FEBID to synthesize (also known as 3D-printing) an artificial twisted nanowire. Right: Experimentally reconstructed spin textures, scanning electron microscopy (SEM) image of the double-helix and micromagnetic simulations showing a complex behavior (*245*).

The scientific and technological exploration of 3D magnetic nanostructures is an emerging research field with exciting novel physical phenomena, originating from the increased complexity in spin textures, topology, and frustration in three dimensions (*239*). 3D nanomagnetism can also be explored using nonflat energy landscapes, where the local curvature affects physical properties across multiple length scales, ranging from the macroscopic to the nanoscale at interfaces and inhomogeneities in materials with structural, chemical, electronic, and magnetic short-range order (*240*). Advanced synthesis, theory, and simulation but specifically experimental approaches to imaging nanoscale spin textures in 3D are essential. Tomographic approaches with magnetic x-ray microscopy are seen as powerful tools and therefore are rapidly being developed and used. The concept is to reconstruct computationally the 3D spin arrangements from an angular series of 2D projection images of magnetic domains. Since magnetization is a vector quantity, typically two angular datasets from rotations around two orthogonal axes are required for high-quality and unambiguous reconstruction. Pioneering work by Donnelly *et al.* (*241*) with hard x-ray ptychography was able to determine the spin textures in the vicinity of Bloch points in micronsized GdFe system. Streubel *et al.* (*242*) reported the first tomographic reconstruction of the spin textures in a strain engineered rolled-up film of Ni. Over the recent past, using focused electron beam–induced deposition (FEBID) (*243*) and two-photon lithography (*244*), a plethora of artificial 3D magnetic nanostructures have been fabricated and imaged with magnetic x-ray nanotomography techniques, including twisted nanowires (*245*) (Fig. 10), helical interconnectors (*246*), bucky-ball structures (*247*), 3D ASI (*248*), and many more.

One of the most recent developments is aiming toward 4D x-ray microscopy, i.e., time-resolved 3D x-ray nanotomography of magnetic nanostructures. In pioneering work by Donnelly *et al.* (*249*), they imaged the dynamic response to a 500-MHz magnetic field of the complex 3D magnetization in a two-phase bulk magnet with a lateral spatial resolution of 50 nm, showing the propagation of a domain wall across a 1.2-μm-thick GdCo_2_ micropillar.

### Frontiers with magnetic x-ray microspectroscopies

The future directions for investigations of magnetic materials and their underlying spin texture with x-rays will tremendously benefit from the laser-like, fully coherent x-ray sources at the next generation of SR facilities. Techniques based on x-ray scattering will tremendously benefit, and, therefore, the characterization of magnetism at fundamental length and timescales will flourish. A particular new x-ray scattering approach is x-ray photocorrelation spectroscopy (XPCS) (*250*), which will allow access to study nanoscale spin fluctuations. First investigations have already been reported. For example, a recent study by Seaberg *et al.* (*251*) used a novel two-pulse XPCS scheme at the Linear Coherent Light Source at the Stanford Linear Accelerator to observe a significant change in the inherent fluctuations at the nanoscale and at picosecond timescales at the transition from a stripe phase to a skyrmion lattice phase in an amorphous GdFe multilayer film (*251*). Although the underlying scientific principles are still under debate, experimental evidence is unquestioned and might open novel ways to control and tailor nanoscale spin fluctuations.

Another frontier at fully coherent x-ray sources is the ability to create and harness x-ray beams with large angular orbital momentum (OAM beams) (*252*, *253*). X-rays with circular polarization can only carry 1ℏ per photon with values of ±ℏ, reflecting right and left circular polarization, whereas OAM x-ray beams will carry more than 1ℏ per photon. The interaction of those OAM x-ray beams with chiral systems, specifically their magnetic properties, will be significantly modified but might also open novel ways of controlling those. Magnetic x-ray spectroscopies and scattering approaches will be most appropriate to explore those OAM beams. The impact to magnetic x-ray microscopies is less obvious at the moment since an OAM beam has a null at its center.

Investigations of magnetic materials with x-rays at those future facilities will have to manage huge data rates, which makes the use of artificial intelligence (AI)/machine learning supported analysis and automated experimental processes indispensable, but will also accelerate the discoveries and understanding of novel magnetic materials, their properties, and behavior, as well as their exploitation toward novel technologies.

## MAGNETISM AT ULTRAFAST TIMESCALES

### Nonthermal laser–induced spin reorientation and spin-phonon coupling pathways for ultrafast magnetic switching

Since spin quantifies the intrinsic angular momentum of a quantum particle, spin dynamics does naturally obey the fundamental law of conservation of angular momentum. For instance, a ferromagnet of volume *V*, where the exchange interaction aligns spins $S_{i}$ and, thus, results in a net magnetization $M=-\gamma \sum_{i}\langle S_{i}\rangle /V$, any change of the magnetization M, is only possible under an external torque T or via angular momentum transfer from or to the spin system. The Einstein–de Haas effect, found back in 1915, showed that a change in magnetization of an object causes a mechanical rotation of the latter and, thus, demonstrated that the lattice is a reservoir of angular momentum. Hence, the efficiency of spin-lattice interactions represents a natural constraint on the speed with which the magnetization can change. Ultrafast magnetism is a field of modern research, which aims to understand spin dynamics triggered by ultrashort (sub–100 ps) stimuli of magnets. These stimuli bring the magnetic media into strongly nonequilibrium states, where additional channels of angular momentum transfer can open up and spin dynamics can often become unexpectedly fast and counterintuitive, thereby forming a challenge for modern theories in magnetism.

An ultrashort laser pulse is known as the shortest possible stimulus in experimental physics of condensed matter and naturally is the main tool in experimental studies of ultrafast magnetism. Already at the early stage of ultrafast magnetism, it was realized that any realistic laser excitation cannot provide enough angular momentum to cause significant changes of the magnetic state as a result of angular momentum transfer from light to matter (*254*). Consider for example ferromagnetic iron, the element from which ferromagnetism derives its very name [ferrum (Latin)]. A volume of 1 cm^3^ contains roughly 10^23^ atoms. If we take photons in the visible spectral range with energies of about 1 eV and assuming that one absorbed photon reverses the spin of a single atom, then the total energy of the photons needed to be absorbed to reverse the net magnetization is about 16 kJ. Taking into account the heat capacity of Fe [3.5 J/(cm^3^·K)], this absorption will lead to an increase of temperature by 5000 K, which is far above its Curie point and even its melting temperature. Hence, understanding the peculiarities of spin-lattice interaction under ultrafast laser excitation is the key for understanding ultrafast magnetism.

In theory, light can induce either reversible or irreversible angular momentum transfer between the lattice and the spins. If the transfer of angular momentum is accompanied by heat exchange, then the process is inherently irreversible. Experimentally, this transfer typically results from laser-induced heating and is therefore referred to as a thermal process, emphasizing that the relevant phenomena can be understood in terms of thermal physics. Conversely, laser-induced spin dynamics that does not depend on heating corresponds to reversible spin-lattice angular momentum transfer and is termed nonthermal.

The possibility of laser-induced spin-lattice angular momentum transfer was recognized shortly after the invention of lasers and the emergence of nonlinear optics. We consider light-matter interaction from a thermodynamic perspective under the assumption that all considered processes are reversible. Under the approximation that light interacts predominantly with electric dipoles and the interaction is linear, the internal energy of the light-matter system is

$$U=U_{0}+\frac{1}{2}\epsilon_{0}\epsilon_{\mathrm{kl}}E_{k}E_{l}^{*}$$ (5)

where $U_{0}$ is the light-independent part of the internal energy, $\epsilon_{0}$ is a fundamental constant, $\epsilon_{\mathrm{kl}}$ is the tensor of dielectric permittivity, $E_{k}$ is the *k*-component of the electric field of light, and $E_{l}^{*}$ is the complex conjugate of the *l*th component of the electric field of light. Since a plethora of magneto-optical effects in magnets demonstrate that $\partial \epsilon_{\mathrm{kl}}/\partial M\neq 0$ while within thermodynamics, the effective magnetic field acting on the magnetization is $H_{\text{eff}}=-\left(1/\mu_{0}\right)\left(\partial U/\partial M\right)$, light can consequently generate an effective magnetic field

$$H_{\text{eff}}=-\frac{1}{2}\frac{\epsilon_{0}}{\mu_{0}}\frac{\partial \epsilon_{\mathrm{kl}}}{\partial M}E_{k}E_{l}^{*}$$ (6)

and thus generate a torque acting on the magnetization

$$T=M\times H_{\text{eff}}$$ (7)

Briefly, optical properties of media do depend on the magnetization $\partial \epsilon_{\mathrm{kl}}/\partial M\neq 0$, and this dependence results in magneto-optical Faraday, Kerr, and Cotton-Mouton effects. Because of the very same dependence, light can affect magnetization via the inverse opto-magnetic Faraday, Kerr, and Cotton-Mouton effects. Since we assumed that the processes are reversible and that no irreversible energy transfer occurs between light and matter, the polarization of light remains unchanged. Consequently, the total angular momentum carried by the interacting photons is conserved. This implies that the spin dynamics driven by the optically induced torque must be accompanied by a reversible transfer of angular momentum between the lattice and the spins.

**Fig. 11.**
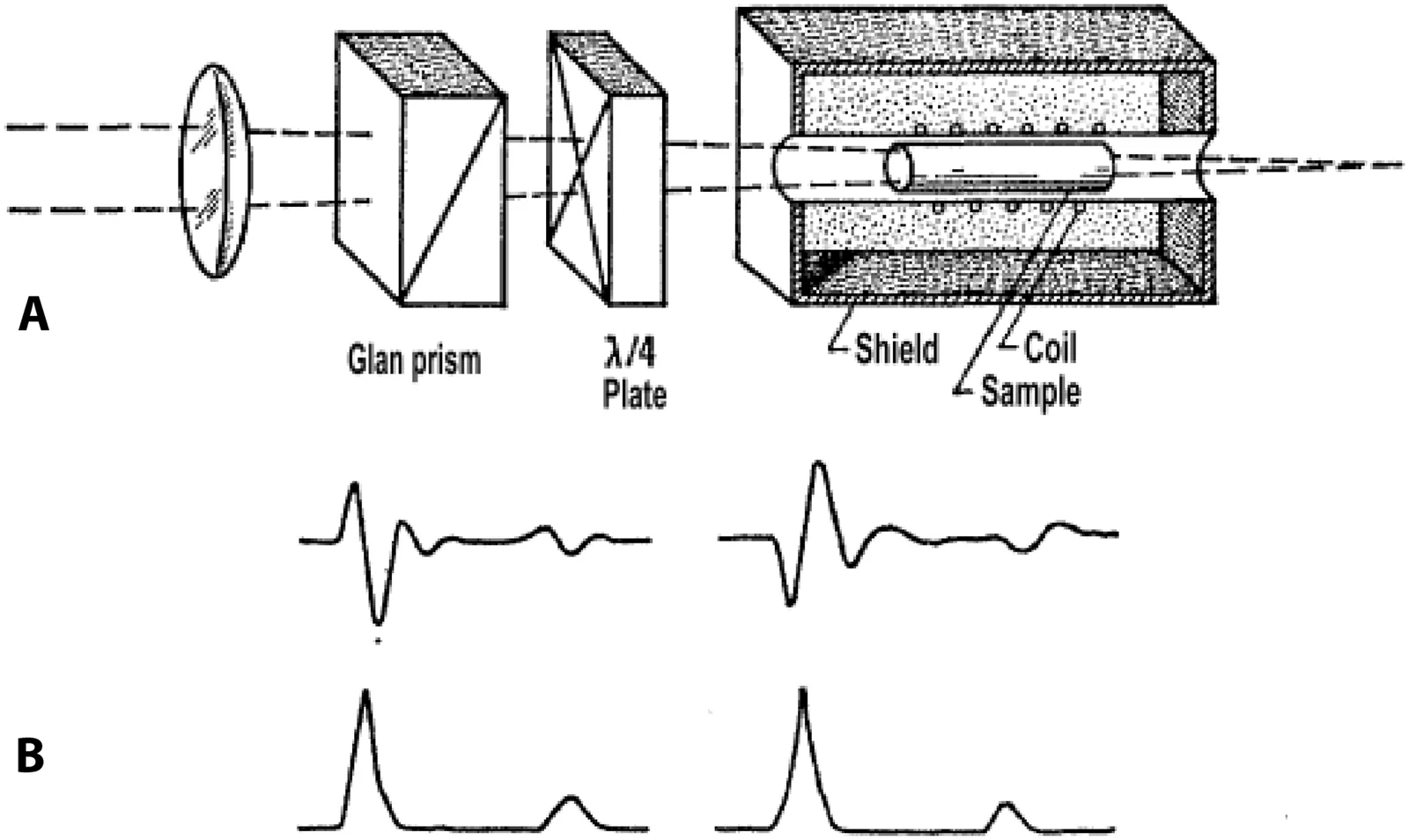
Inverse Faraday effect. (**A**) Schematic illustration of the experimental geometry for observation of the inverse Faraday effect. (**B**) Typical signals of the inverse Faraday effect (top) and laser intensities (bottom) observed in Eu-doped CaF_2_ at 4.2 K under pumping the medium with circularly polarized light. Reprinted with permission from (*256*). Copyright (1966) by the American Physical Society.

The idea of the inverse opto-magnetic Faraday effect was first proposed by Pitaevskii (*255*). Soon, a similar phenomenon was independently proposed and studied experimentally by van der Ziel and colleagues (*256*, *257*). The experimental results are shown in Fig. 11, where an initially linearly polarized laser pulse first acquires a circular polarization using a quarter wave plate. Afterward, the laser pulses interact with the sample—a rod of paramagnetic Eu-doped CaF_2_, placed in the center of a coil. The intensity of light within the rod correlated with the electrical signals induced in the coil, demonstrating magnetic changes in the sample.

Stimulated Raman scattering and the inverse Faraday effect are closely related processes. Describing the interaction of light with magnets in the time domain and accounting for the inverse Faraday effect, the effective magnetic field generated by light will trigger oscillations of spins. In the frequency domain, this corresponds to the generation of spin waves and corresponds to Raman scattering of light by spin waves.

The invention of femtosecond lasers and the emergence of ultrafast magnetism led to the question about the feasibility of the inverse Faraday effect on the femtosecond timescale. In 2005 Kimel *et al.* (*258*) showed that circularly polarized femtosecond laser pulses can impulsively excite spin resonances in antiferromagnetic DyFeO_3_, demonstrating that the generated pulses of the effective magnetic field pulses are equally short. Changing the helicity or propagation direction of the pulses, it was possible to control the phase of the oscillations or address a particular mode of spin resonance in a full agreement with the theory of the inverse Faraday effect. Afterward, femtosecond inverse Faraday effects and Cotton-Mouton effects have been reported in many magnetic materials, but it is remarkable that most of those are not ferromagnets but ferri- and antiferromagnets. This fact is easy to understand whether one remembers that spin dynamics must obey the law of conservation of angular momentum and any substantial changes of magnetization and, thus, changes of angular momentum must be accommodated by the lattice. For this reason, it is easier to launch large amplitude spin dynamics in materials with a small, vanishingly small or even zero magnetization. Ferri- and antiferromagnets are thus the most promising materials for an efficient and ultrafast control of magnetism using light.

**Fig. 12.**
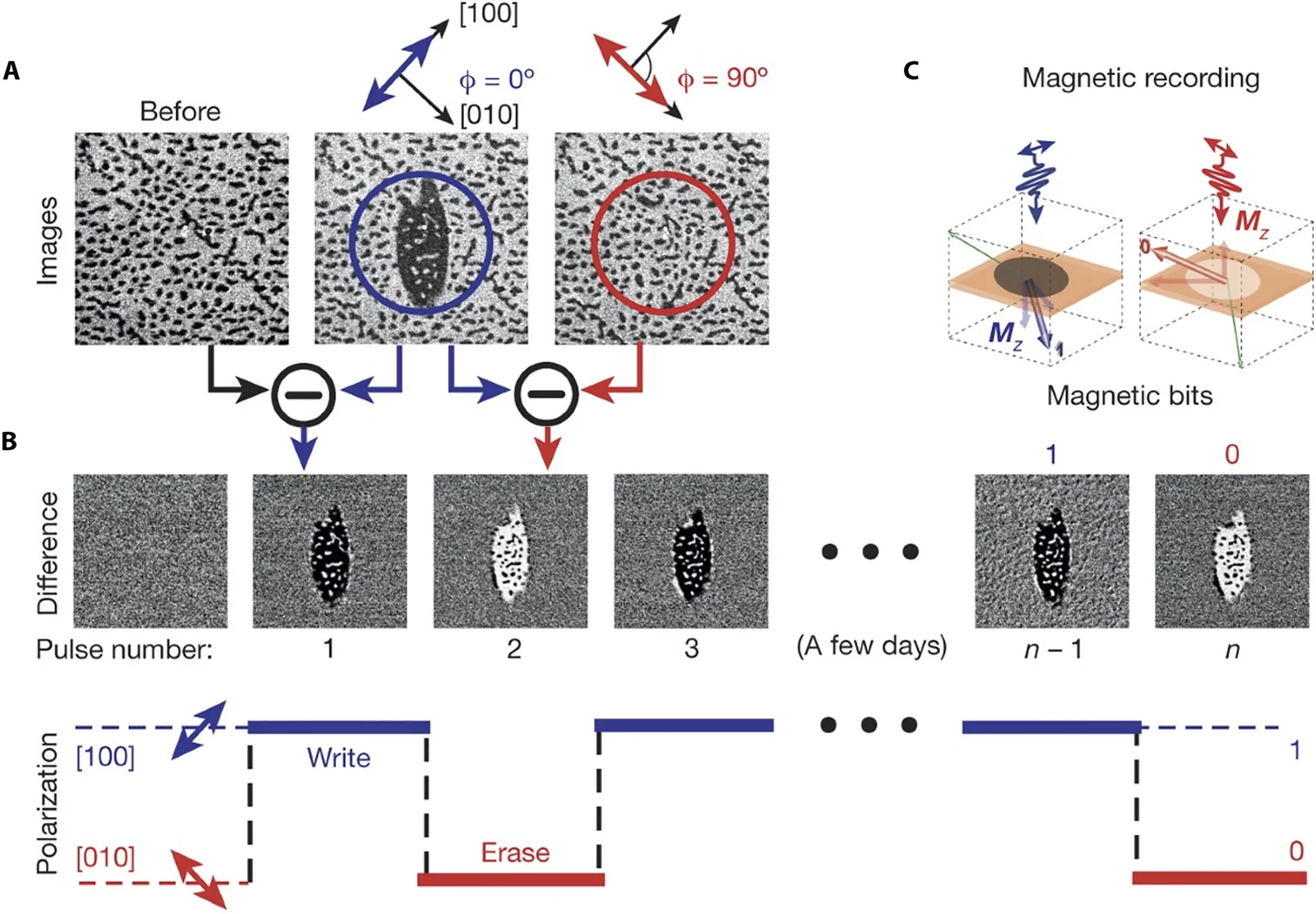
Ultrafast all-optical magnetic recording on transparent iron garnet using linearly polarized light. (**A**) Images before laser excitation and after excitation with linearly polarized pulses with two mutually orthogonal linear polarizations. (**B**) Differential images demonstrating robust writing of magnetic bits. (**C**) Pictorial diagram showing magnetic switching between two stable states (*259*).

In 2017, Stupakiewicz *et al.* (*259*) demonstrated a breakthrough in magnetic data writing (see Fig. 12): An all-optical, ultrafast magnetic recording in transparent dielectric films—specifically, cobalt-substituted garnet—achieved with negligible heat load. Using a single femtosecond laser pulse with linearly polarized light, specific d-d electronic transitions within cobalt ions were targeted. This resonant excitation transiently alters the magnetic anisotropy, breaking the energetic degeneracy between two magnetic metastable states. By selecting the laser’s polarization direction, the net magnetization could deterministically be switched to write a “0” or “1” bit. This enabled ultrafast writing with remarkable efficiency—the entire write-read event completes in under 20 ps, and the heat injected into the medium remains extremely low—below 6 J/cm^3^. In essence, the study establishes a nonthermal, light-driven magnetization control scheme in garnet films that is both ultrafast and energy efficient, pointing toward the future of optical magnetic memory devices.

Very recently, it was demonstrated that the combined action of the thermal effects of light in a magnetic GdFeCo film and nonthermal effects in the crystalline substrate can also facilitate the writing of magnetic bits with light in the mid-infrared spectral range (*260*). By pumping phononic resonances in the substrate, it was possible to steer the process of spin reversal in the magnetic film in the direction defined by the helicity of the phonons. This finding opens up a new chapter in optical control of magnetism and, in particular, in heterostructures engineered to maximize specific effects of spin-lattice interaction.

### Femtomagnetism and subpicosecond AOS of spins

The interaction of light with magnetic matter is of both fundamental and practical interest: Already since the discovery of the magneto-optical effect by Faraday in 1846 (*192*), later experimental studies by Zeeman in 1897 (*261*) and the theory by H. Lorentz in 1897 (*262*), we know that magnetic order can affect light. Not only these developments have led to magneto-optical spectroscopy using the Faraday, Kerr, and MCD and related effects, but also applications such as Faraday isolators, magnetic resonance imaging, and measurements of magnetic fields of distant stars. Their inverse (Inverse Faraday) effects have been discussed in the “Nonthermal laser–induced spin reorientation and spin-phonon coupling pathways for ultrafast magnetic switching” section.

Using modern femtosecond lasers, we can probe magnetization dynamics down to its fundamental timescale, while the inverse effects are equally interesting and potentially useful (see description about nonthermal effects). More particularly, since the seminal work of Beaurepaire *et al.* (*263*), we know that femtosecond laser excitation of metallic ferromagnets can lead to an ultrafast, i.e., subpicosecond, demagnetization on a timescale much faster than any of the interactions at which spins can exchange energy or angular momentum near thermodynamic equilibrium. The authors described their unexpected result using a phenomenological three-temperature model in which, first, the laser energy is absorbed by the free electrons so that light energy turns into heat on the timescale of the laser pulse. Subsequently, energy and angular momentum are exchanged between the electron, phonon, and spin reservoirs, described by electron-phonon (2 ps) and spin-lattice (100 ps) relaxation parameters. The data could be well described by introducing an additional phenomenological relaxation parameter characterizing electron-spin interaction (1 ps). Aiming to understand the physical origin and microscopic physical mechanisms defining this phenomenological parameter has been a challenge and motivated numerous fundamental studies of ultrafast demagnetization of ferromagnetic metals during the past three decades (*264*, *265*).

**Fig. 13.**
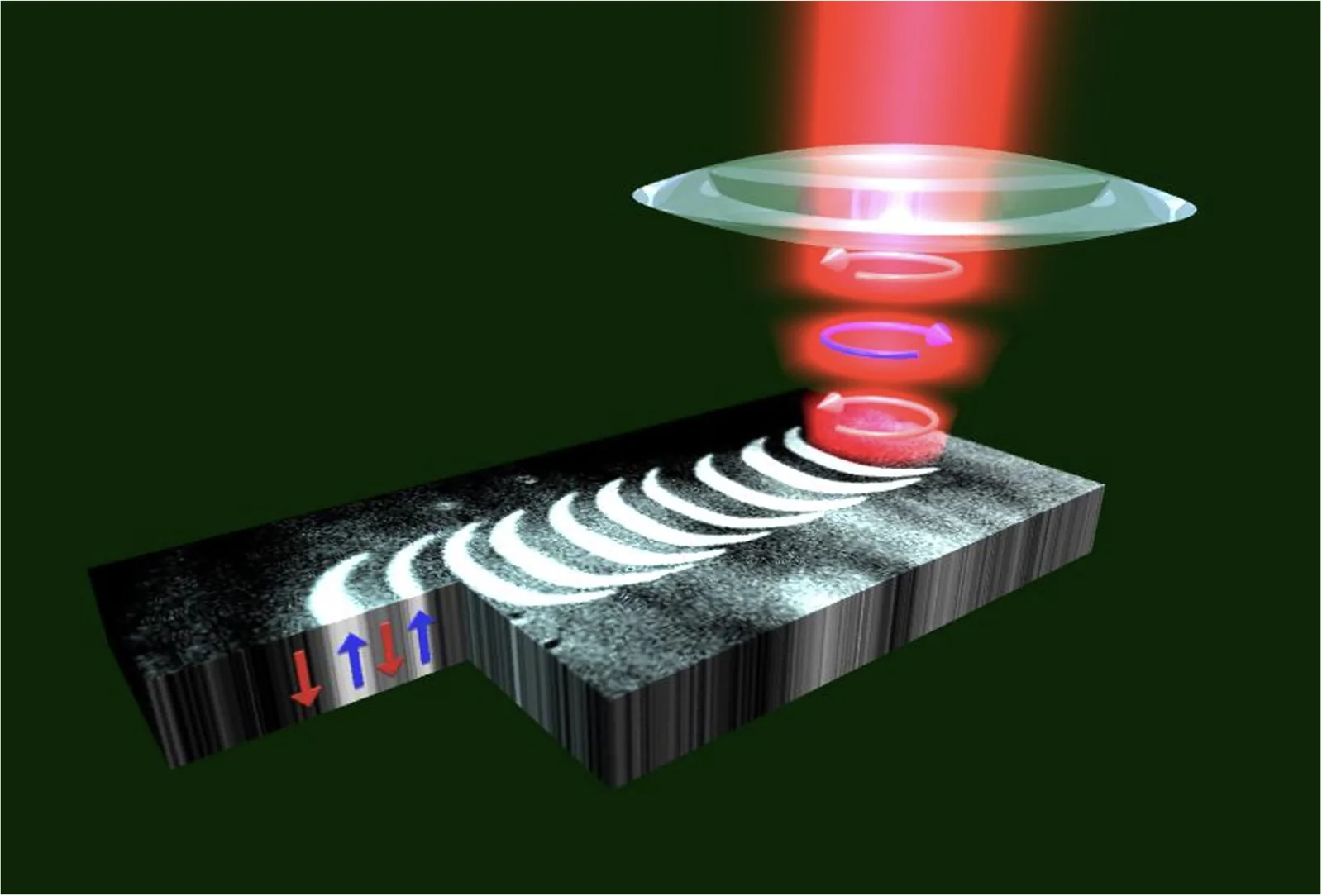
Schematics of AOS. AOS of magnetization, by moving a 40-fs, circularly polarized, laser beam with a wavelength of 800 mm and a fluence around 1 mJ/cm^2^ over a thin FeGdCo film while switching the helicity between successive pulses. Reprinted with permission from (*266*). Copyright (2007) by the American Physical Society.

Fundamental interest in ultrafast magnetism soon included ferri- and antiferromagnetic materials. Ferrimagnetic materials, in this respect, have shown so far the most marked response to ultrafast laser excitation, starting with the 2007 observation of AOS of the magnetization in the ferrimagnetic metallic alloy GdFeCo with a single circularly polarized 40-fs laser pulse, where the final state of the magnetization was defined by the helicity of light (see Fig. 13) (*266*).

The counterintuitive nature of femtosecond laser–driven switching of magnetization becomes evident when considering the extreme effective magnetic fields required. Rotating a free electron spin by 180° within 100 fs would require a precessional frequency of 5 THz and an effective field of 100 T, comparable to the exchange field of spin-spin coupling.

**Fig. 14.**
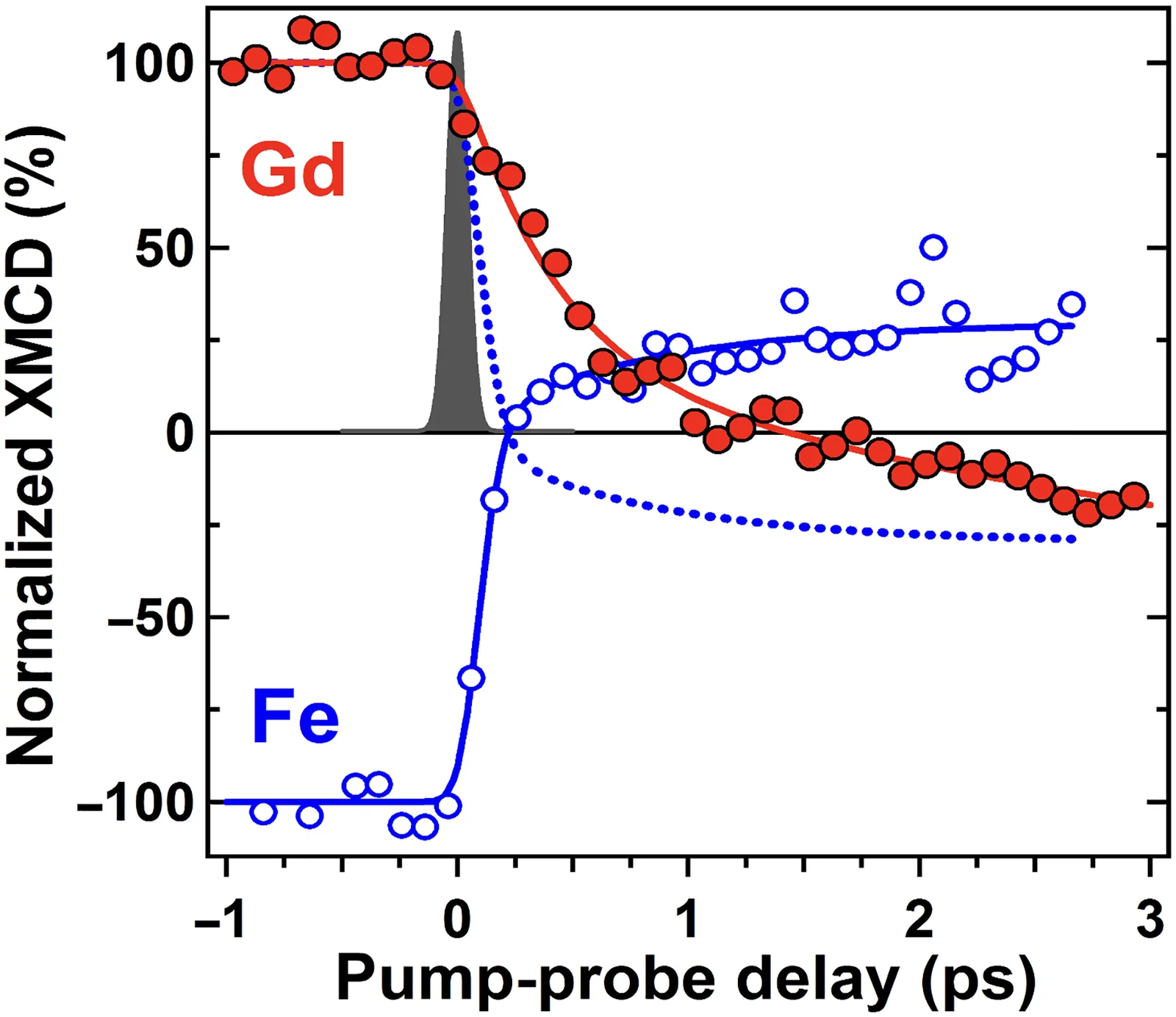
Element-resolved dynamics of the Fe and Gd magnetic moments in a FeGdCo thin film. Measured by time-resolved XMCD using circularly polarized 100-fs x-ray pulses centered at the Fe *L*_3_ edge and Gd *M*_5_ edge, respectively, with femtosecond time resolution using the slicing beam at BESSY II in Berlin. The film was excited with a 60-fs laser pulse at 800 nm. Reproduced from (*269*).

To describe AOS, early model assumed identical dynamics for both magnetic sublattices of GdFeCo (i.e., Gd and FeCo), effectively treating the material as a single-spin system, while the laser pulse acted as an ultrafast heat source for electrons and as an effective magnetic field via the inverse Faraday effect. These models reproduced the experimental Fe-spin dynamics but required unrealistically large opto-magnetic fields (>20 T) and prolonged pulse residence times, leaving the role of Gd spins unresolved. However, Radu *et al.* (*267*) experimentally showed that demagnetization rates scale inversely with the magnetic moment while in the case of multisublattice systems; this demagnetization rate is also affected by the sign of the sublattice exchange (*267*, *268*). In 2011, however, an unexpected discovery was made: Deterministic AOS of GdFeCo was achieved with a linearly polarized femtosecond laser pulse (*269*). Obtaining a physical picture for this unexpected AOS without an external symmetry breaking stimulus required separately probing the ultrafast dynamics of the individual magnetic sublattices (Gd and Fe, neglecting Co due to its small contribution), which was possible via the element-specific technique of XMCD, which is described in the “X-rays and magnetism” section and the availability of femtosecond x-ray pulses. It was shown that ultrafast laser excitation of GdFeCo gives rise to an ultrafast heating of the GdFeCo sample, decreasing the magnetizations of both sublattices rapidly. However, whereas the magnetization of Fe collapsed within 0.2 ps, the demagnetization of Gd took as long as 1.5 ps (see Fig. 14).

This virtually independent demagnetization dynamics of the two sublattices is remarkable considering the strong antiferromagnetic exchange interaction between them but can be understood from the fact that the Gd moment is about four times larger than that of Fe, leading to a longer demagnetization time (*267*). Subsequently, after the electronic bath cooled down, the continuing slow demagnetization of Gd is accompanied by the growth of the magnetization of Fe in the opposite direction, thus conserving total angular momentum. This resulted in the observed transient ferromagnetic-like state with parallel Fe and Gd magnetizations in the time interval between 0.2 and 1.5 ps after the excitation. Last, after complete demagnetization of the Gd sublattice in the presence of a finite reversed Fe magnetization, the antiferromagnetic exchange interaction drives the Gd sublattice in the direction antiparallel to that of Fe on the picosecond timescale, completing AOS of GdFeCo. A subsequent pulse will again lead to a switching of this ferrimagnetic magnetization, implying that the magnetization can be toggled back and forth between its two states, independent of its starting point. On the basis of the Onsager relations for spin dynamics, Mentink *et al.* (*270*) derived the equations of motion for a system of two nonequivalent collinear sublattices that described the experimentally observed nonequilibrium dynamics very well. A similar picture was found via atomistic simulations by Ostler *et al.* (*271*) and by Evans *et al.* (*272*) for synthetic ferrimagnets. The latter was also observed experimentally (*273*). It was also shown that the observed helicity dependence in GdFeCo (*269*) could be attributed to MCD (*274*), which is consistent with the ultrafast heating mechanism of the descriptions mentioned above.

These results were more recently generalized and resulted in a phase diagram that described the different pathways for AOS, implying that, for proper pulse and sample parameters (pulse width, fluence, and sample composition), a femtosecond or even picosecond excitation pulse can result in the reversal of the original magnetization, independent of the polarization of the pulse (*275*). The key ingredients for successful AOS of ferrimagnets are the differences in the properties of A and B, where A and B are the transition metal ferromagnet and the rare-earth paramagnet in isolation, respectively. $m_{A}<m_{B}$, and the antiferromagnetic exchange coupling between them gives rise to a common Curie temperature in equilibrium and the existence of two degenerate equilibrium states, with A and B having antiparallel magnetization. Switching of this system is achieved when *m*_A_ reaches zero before *m*_B_, that is, the demagnetization rate of A must be higher than that of B. This will work even for picosecond laser pulses, as long as $\tau_{A,B}<\tau_{s-l}$ and the pulse width is shorter than the former. When the laser pulse is much shorter, the dissipation of angular momenta to the external environment overwhelms at first the exchange coupling, but when *m*_A_ crosses zero, the intersublattice exchange finishes the process. When the difference between *m*_A_ and *m*_B_ is larger, there is more angular momentum available to transfer and consequently even longer pulses can achieve switching, as long as the nonadiabatic condition $\tau_{A,B}<\tau_{s-l}$ is fulfilled. Switching with pulses as long as 15 ps has been achieved for a Gd concentration of 27. 5 (*276*), which is in good agreement with the phase diagram of (*275*).

As one of the key ingredients is the nonadiabatic heating of the electronic subsystem, other triggers, such as a short current pulse, can also be used, as was successfully demonstrated for GdFeCo in (*277*). As the heating only involves transfer of energy to the electron subsystem, the specific photon energy is also not relevant, which was confirmed by changing the latter with a factor of more than 20, still seeing AOS as long as the pulse width was in the right regime (*275*).

Single-shot switching was, so far, only observed in a limited number of RE-TM systems such as GdFeCo (*266*), TbFeCo (*278*, *279*), and Gd/Co (*273*) and Tb/Co multilayers (*280*). However, recently, it was observed that single-shot AOS was also achieved in the ferrimagnetic Heusler alloy Mn_2_Ru*_x_*Ga, which could be described by the same exchange-driven mechanism (*281*).

Although plasmonic antennas have allowed to push AOS in ferrimagnets even down to the nanometer length scale (*279*) and photonic networks allow the development of an optically switchable magnetoresistive random access memory (*282*), ferromagnets remain the material of choice for technological applications due to their high anisotropy and ease of production. However, it took many years (and longer laser pulses) before AOS was found in Co/Pt multilayers (*283*) and FePt granular media (*284*). Although AOS in these ferromagnets appeared to be helicity dependent (HD-AOS), which, for deterministic switching, is advantageous, the drawback was that it required many (hundreds) pulses, making the overall switching process very slow. Detailed investigations suggested that HD-AOS proceeds via a two-step process, starting from a helicity independent formation of switched domains while being completed via slow domain wall displacements by many circularly polarized pulses via MCD (*285*). This two-step process led to the idea of dual-pulse AO-HDS to accelerate the AO-HDS in ferromagnets by steering the nanoscale nuclei of correlated spins created in a femtosecond laser–induced strong nonequilibrium state, with a picosecond circularly polarized pulse using MCD (*286*). By properly adjusting the delay between the femtosecond excitation and the picosecond steering pulses, it was found that a single pulse pair consisting of a linearly polarized 90-fs pulse and a 4.5-ps circularly polarized pulse, separated by a 5-ps delay, leads to a switching efficiency of 80%, thereby markedly reducing the time of optical writing of data in Co/Pt (*287*). To understand the spatiotemporal aspects of this ultrafast switching process, an atomistic spin dynamics model was developed. The model describes the production of randomly oriented nanometer-scale spin textures after ultrafast demagnetization due to the first 90-fs π-pulse and the differential (helicity-dependent) heating of the spin textures by the second 4.5-ps σ-pulse via the mechanism of MCD. Atomistic spin dynamics simulations of the dual-pulse AO-HDS reproduce the narrow (5-ps) optimal window of Δt for the spin switching. The time evolution of the mapped electron and spin temperatures shows that the nonequilibrium spin textures created by the femtosecond π-pulse reverse within about 3 ps by the spin- and helicity-dependent temperature difference of only a few kelvin produced by the picosecond σ-pulse, a mechanism circumventing the need for domain wall displacement and operating at the nanoscale (*287*). Very recently, using a magnetic force microscope to investigate the domain formation of the magnetization induced by circularly polarized picosecond laser pulses, Khusyainov *et al.* (*288*) observed the stochastic nucleation of complex nanotextured domains. The growth of these domains appeared to be determined by the complexity of the texture and was in line with the atomistic modeling discussed above. Ultrafast laser excitation can also manipulate nanoscale spinstructures in domain walls, as demonstrated by Pfau *et al.* (*289*) and generate spin-transfer torques, as shown by Schellekens *et al.* (*290*). Femtosecond laser excitation can also be used to create topological protected structures such as skyrmions through a fluctuation-mediated phase, as recently demonstrated by Büttner *et al.* (*291*).

The increasing technological interest in antiferromagnets has also triggered interest in the optical manipulation of antiferromagnetic order by light, particularly because the absence of a net magnetization makes it hard to control antiferromagnetic order otherwise. Using the coupling between magnetic and electric order in the multiferroic TbMnO_3_, optical writing and erasure of antiferromagnetic domains were demonstrated in this material using light pulses of two different colors (*292*). Very recently, the optical switching of antiferromagnetic order in the metallic antiferromagnet Mn_2_Au using laser-induced optical torques was predicted, using atomistic spin dynamics simulations (*293*).

## SUMMARY AND OUTLOOK

One hundred years after the introduction of the concept of the spin of the electron by Goudsmid and Uhlenbeck, research into electron spin has transformed our understanding of magnetism and opened entirely new avenues for materials science and technology. This review has examined the state of the art in probing spin textures across the nanometer-to-micrometer length scales and femtosecond-to-millisecond timescales using neutron scattering, resonant x-ray techniques, magnetic x-ray spectromicroscopy, and ultrafast optical methods, each offering complementary and often irreplaceable windows into magnetic phenomena.

Neutron scattering has proven uniquely powerful for characterizing both static and dynamic spin correlations across a broad range of energy and length scales. Landmark experiments have elucidated complex magnetic structures—from incommensurate spin textures in multiferroic TbMnO_3_ and skyrmion lattices in MnSi to exotic quantum states such as spin ice and fractionalized spinon excitations in low-dimensional and frustrated magnets. The ability to probe driven, out-of-equilibrium spin systems through time-resolved techniques—using pulsed magnetic fields, resonant microwaves, and laser excitation—is opening an exciting new frontier for understanding spin dynamics on timescales inaccessible to conventional inelastic scattering approaches.

RXS techniques, including REXS and RIXS, have emerged as indispensable complements to neutron methods, enabling momentum-resolved studies of magnetic, orbital, and charge order in systems where neutrons are impractical. These tools have been central to establishing the spin-orbit–entangled electronic ground states of iridates and ruthenates, revealing the physics of Kitaev magnets and quantum spin liquids and mapping quantum critical fluctuations in correlated electron systems from cuprate superconductors to metamagnetic ruthenates. Continued advances in energy resolution—now approaching ≈10 meV at soft x-ray and below ≈5 meV at hard x-ray RIXS spectrometers—are steadily closing the gap with neutron spectroscopy while offering the additional advantages of element selectivity and compatibility with thin-film and small-crystal samples.

Magnetic x-ray spectromicroscopy has evolved markedly since the first magnetic domain images obtained in the early 1990s, now encompassing a rich suite of real-space and reciprocal-space imaging techniques including X-PEEM, MTXM, STXM, coherent diffractive imaging, ptychography, holography, and laminography. These methods have illuminated the static and dynamic behavior of topological spin textures such as skyrmions and Hopfions and are advancing rapidly toward full 4D (3D + time) nanotomographic imaging of magnetization dynamics. The transition to fourth-generation diffraction-limited storage rings and x-ray free electron lasers will provide fully coherent x-ray beams that promise transformative new capabilities, including x-ray photon correlation spectroscopy for accessing nanoscale spin fluctuations and orbital angular momentum beams for probing chiral magnetic systems in entirely new ways.

At ultrafast timescales, femtosecond laser experiments have revealed that spin systems can be driven into strongly nonequilibrium states where angular momentum transfer proceeds through unexpected pathways, enabling subpicosecond demagnetization, all-optical helicity-dependent switching in ferrimagnets, and nonthermal photomagnetic recording in insulating garnets. The discovery of exchange-driven AOS in rare-earth/transition-metal ferrimagnets has stimulated broad interest in ultrafast spin control, and recent demonstrations of phonon-mediated spin reversal via mid-infrared excitation are opening new routes to engineer spin-lattice coupling in heterostructures.

Looking ahead, the convergence of these experimental capabilities with emerging material platforms—including antiferromagnetic and altermagnetic van der Waals systems, artificially designed 3D magnetic nanostructures, and topological magnetic textures such as Hopfions—promises to enrich the field substantially. New functional paradigms, including spin-phonon coupling as a handle for ultrafast control and neuromorphic-like computing schemes that exploit spin coherence and correlations, are beginning to be explored. AI- and machine learning–assisted data analysis will be essential for managing the enormous data volumes generated at next-generation facilities and for accelerating the discovery and understanding of novel magnetic materials. One hundred years after the prediction of the electron spin, its role in fundamental science and transformative technology continues to deepen, and the coming decade of experimental advances with neutrons, x-rays, and optical probes is poised to yield discoveries of comparable significance to those that have defined the field’s remarkable history.
